# Bimodal sensing of guidance cues in mechanically distinct microenvironments

**DOI:** 10.1038/s41467-018-07290-y

**Published:** 2018-11-20

**Authors:** Erdem D. Tabdanov, Vikram V. Puram, Zaw Win, Ashab Alamgir, Patrick W. Alford, Paolo P. Provenzano

**Affiliations:** 1 Department of Biomedical Engineering, University of Minnesota, Minneapolis, MN USA; 2 University of Minnesota Physical Sciences in Oncology Center, Minneapolis, MN USA; 3 Department of Biological Engineering, Cornell University, Ithaca, NY USA; 4 Masonic Cancer Center, University of Minnesota, Minneapolis, MN USA; 5 Stem Cell Institute, University of Minnesota, Minneapolis, MN USA; 6 Institute for Engineering in Medicine, University of Minnesota, Minneapolis, MN USA

## Abstract

Contact guidance due to extracellular matrix architecture is a key regulator of carcinoma invasion and metastasis, yet our understanding of how cells sense guidance cues is limited. Here, using a platform with variable stiffness that facilitates uniaxial or biaxial matrix cues, or competing E-cadherin adhesions, we demonstrate distinct mechanoresponsive behavior. Through disruption of traction forces, we observe a profound phenotypic shift towards a mode of dendritic protrusion and identify bimodal processes that govern guidance sensing. In contractile cells, guidance sensing is strongly dependent on formins and FAK signaling and can be perturbed by disrupting microtubule dynamics, while low traction conditions initiate fluidic-like dendritic protrusions that are dependent on Arp2/3. Concomitant disruption of these bimodal mechanisms completely abrogates the contact guidance response. Thus, guidance sensing in carcinoma cells depends on both environment architecture and mechanical properties and targeting the bimodal responses may provide a rational strategy for disrupting metastatic behavior.

## Abstract (web summary)

Invasive cells respond to contact guidance cues during migration. Here, using micro- and nanopatterning with different ligands and varying stiffness, the authors find that cells can make cellular protrusions through both contractility-dependent and contractility-independent means.

## Introduction

Directed cell migration is essential for numerous physiological processes, such as embryonic development, immune function, and tissue repair^1–3^. It is also essential for cancer invasion and ultimately metastatic dissemination^4–6^. In particular, carcinoma cell migration along aligned stromal collagen is known to facilitate directed migration through contact guidance that leads to poor outcomes in human patients^7–10^, yet our understanding of the impact of fiber stiffness and the molecular mechanisms governing carcinoma cell contact guidance remains quite limited. In an effort to elucidate the physical and molecular mechanisms governing contact guidance of carcinoma cells, a number of powerful engineered two- and three-dimensional (2D and 3D, respectively) platforms have been employed to recapitulate directed motility behavior, including 2D substrates with nanoscale and/or microscale features mimicking stromal collagen extracellular matrix (ECM)^11–13^ and aligned 3D collagen matrices^9,11,14^. From these fundamental works, a number of key questions have emerged, including whether or not cell and matrix mechanics and forces across cell–cell interactions impact contact guidance. Indeed, recent studies have suggested that the mechanical properties of the microenvironment may regulate carcinoma cell contact guidance^11,12^, while separate work has suggested that distinct, opposing responses to contact guidance can emerge depending on whether carcinoma cell traction forces are reduced by targeting intrinsic actomyosin contractility or by decreasing ECM stiffness^15^. As such, questions remain as to whether the effective traction within the cell–ECM linkage, generated via cell-intrinsic traction (i.e., actomyosin contractility) or signaling feedback regulated by ECM mechanics, have synergistic or antagonistic effects on contact guidance. This is due, in part, to a lack of engineered systems that incorporate defined ECM alignment on substrates of variable stiffness to parse out the influence of the mechanical properties of guidance cues. Furthermore, to date, systems have also not allowed for spatial separation of cell–ECM versus cell–cell adhesion systems, which can provide competing or coordinated cues that influence cellular sensing of contact guidance cues^11,16–18^. Thus here we generated platforms that overcome these deficits in order to dissect the physical and molecular mechanisms governing sensing of contact guidance cues.

There are two dominant cell adhesion systems that regulate cell–ECM or cell–cell interactions, namely, integrins and cadherins^17,19,20^. Integrin-mediated adhesion is a dominant regulator of cell motility and is particularly relevant for carcinoma cell migration in 3D tumor ECMs that include a robust collagen network^5,21,22^. These transmembrane receptors act to transmit forces between the extracellular environment and the actin cytoskeleton while also participating in robust signaling activity through focal adhesions (FAs) to regulate essential cell functions, such as proliferation and migration^20,23^. Furthermore, changes in actin structure and dynamics are known to influence FA signaling and cell protrusion dynamics and are regulated by a number of signaling processes, including Arp2/3-dependent actin branching that can regulate protrusion dynamics and formins that are required for effective traction force transmission between FAs and the actin cytoskeleton^24–28^. Conversely, cell–cell adhesion is largely regulated by E-cadherin in epithelial cells^19,29^ and its loss can trigger a critical switch that drives aggressive single-cell migration following epithelial-to-mesenchymal transition^6,30^. Furthermore, like integrins, forces are transmitted across E-cadherins where they act as a scaffold between the actin cytoskeleton and receptors on adjacent cells to induce strong normal forces across cell–cell adhesions^18,19^. Indeed, we recently reported that forces resulting from cell–cell interactions diminish contact guidance by competing with anisotropic forces that result from aligned ECM^11^. Thus, separating out the relative impact of these adhesion systems, and their associated mechanics and mechanotransduction, is complex and requires specifically engineering platforms to isolate the factors driving fundamental cell behaviors.

Here, to address fundamental questions surrounding cancer cell sensing and response to contact guidance, we engineered ligand-patterned surfaces with specific architectures and tunable mechanical properties that allow adhesion via cell–matrix and/or synthetic cell–cell interactions (i.e., type-I collagen and E-cadherin ligands). Using these platforms, we demonstrate that carcinoma cells possess two distinct modes for sensing guidance cues. The first mechanism is contractility-dependent, utilizing conventional myosin-dependent traction stress through FAs and actin stress-fibers, and requires formins and focal adhesion kinase (FAK) activity. The alternate contractility-independent mechanism emerges from a distinct switch toward a more phenotypically dendritic, fluidic-like cell state in which protrusive dynamics are no longer sensitive to the surrounding matrix mechanics but are driven by Arp2/3-dependent branched actin networks and guided by ligand topography. Although both subsystems share certain common features, such as contact guidance, the mechanical aspects of their function are fundamentally different, and concurrent disruption of these bimodal mechanisms completely abrogates the response to contact guidance. Thus these mechanisms co-exist and we demonstrate that they can work side-by-side as well as independently in order to regulate the bimodal mechanisms of contact guidance sensing.

## Results

### Engineering the microenvironment to quantify guidance sensing

**Fig. 1:**
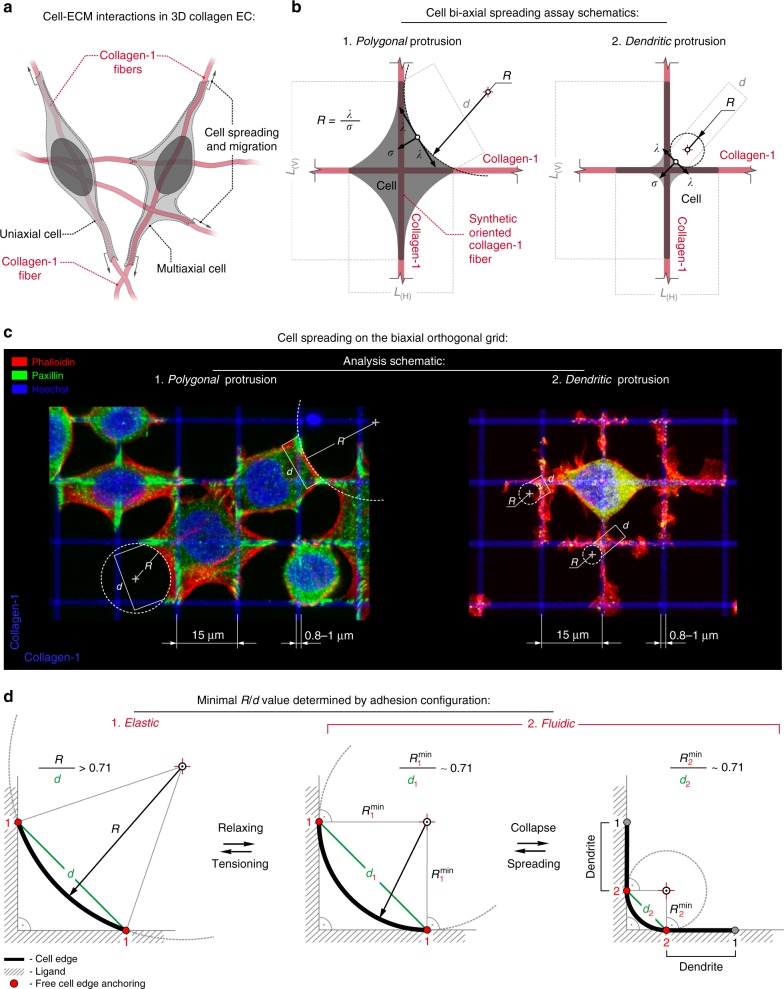
Biomimetic platform to define directional and mechanical guidance cues. **a** Schematic of prominent cell adhesion interactions in 3D fibrous microenvironments. **b** Schematic of the range of cellular responses to biaxial cues from orthogonally oriented type I collagen lines. See also Supplementary Figure 1 that describes the uniaxial guidance cues employed in this study, which represent anisotropic oriented collagen fibers versus biaxial cues that model non-oriented fibrous matrices. We note that both of these are distinct from quasi-2D conditions that result from closely spaced nanolines. To quantify the mechanical response to soft and stiff substrates, we employ a 2D Laplace law model that describes the relationship between cell shape and cell tension. In the Laplace model, free cell edge curvature radii reflect the balance between the internal tension *σ* and linear cell edge tension *λ* following *R* = *λ*/*σ*, where larger *R* correlates with greater forces at adhesion sites. **c** Examples of distinct cell architectures (3D reconstructions), corresponding to the schematics outlined in **b** that we show here can emerge in response to biaxial guidance cues and specific mechanical relationships between the cell and matrix. For analysis of cell mechanics with a 2D Laplace law formulation, the orthogonal guidance cues determine the minimal *R*/*d* value (~0.71) for any spanning distance *d*. For more dominantly elastic behavior, *R*/*d* is >0.71 and a polygonal phenotype dominates. In contrast, instability emerges as *R*/*d* is at or near the minimum value where protrusions collapse onto the orthogonal lines resulting in more fluidic-like protrusion behavior with a dendritic phenotype

A powerful way to mimic key aspects of aligned in vivo ECM architecture and dissect the governing mechanisms is to generate ECM anisotropy while retaining the advantages of 2D systems, which allows for quantitative analysis of large cell numbers under defined conditions. Here we impart guidance cues by patterning collagen to represent size-relevant aligned fibers (0.8–1 µm) on substrates with definable mechanical properties in order to parse out physical and molecular mechanisms governing guidance sensing. As the basis for our platform, we employed 1D or “uni-dimensional” adhesion that has been shown to represent key aspects of 3D motility^31^ and 2D biaxial systems to mimic multidirectional cues from physiologically relevant fibrous networks, which are distinct from planar 2D adhesion surfaces and tightly packed nanolines that represent a quasi-2D interaction (Fig. 1a, b, Supplementary Figure 1). These ECM patterns represent anisotropically oriented fibers at the scale of single-cell interactions with the matrix but with the advance of patterning on physiologically relevant soft (*G*’ = 2.3 kPa) and stiff (*G*’ = 50 kPa) surfaces^32^, which are distinct from fundamental work varying stiffness of fibronectin-coated microchannels that induce cell confinement^33^ as opposed to contact guidance. Thus our platforms allow us to test the influence of both directional and mechanical properties of the microenvironment during sensing of aligned ECM, which has remained elusive to date (Fig. 1a–c, Supplementary Figure 1). Further, precisely patterning collagen, which is a dominant ECM guidance cue in breast and pancreatic carcinomas^7–11,34^, has been historically difficult, in part due to its propensity to undergo gelation; therefore, it is frequently replaced with fibronectin. Here we employ a method we developed for uniform submicron-level patterning with gentle cross-linking of type-I collagen to polyacrylamide (PAA) gels that allowed us to evaluate both mechanical and topographical interplay from collagen guidance cues.

To evaluate cell mechanics during the response to guidance cues, we employed a 2D Laplace’s law model that describes the relationship between cell shape and tension and has been shown to accurately predict force at adhesion sites^35–37^. We note that conventional traction force microscopy (TFM) provides great utility to quantify cell traction and has been used extensively by us, e.g., refs. ^11,38^, and numerous others, e.g. refs. ^32,39,40^, to capture fundamental cell traction force behavior. However, depending on the magnitude of cell traction, TFM can fail to allow accurate traction measures on higher stiffness substrates^32^. Indeed, our analysis of traction forces on soft and stiff environments likewise demonstrates that traction forces are not of sufficient magnitude in the breast carcinoma cells used in this study to create accurately measurable displacements on stiff substrates (Supplementary Figure 2). As such, we employed and expanded upon an established alternate approach to quantify cell mechanics that also readily allows for higher-throughput quantification of large numbers of cells compared to classical TFM. Here the grid architecture of our platform facilitates the formation of free cell edges between two adjacent orthogonal lines (Fig. 1, Supplementary Figure 1). Quantifying the free cell edge radii, *R*, provides a metric of linear tension (*λ*) in the arc relative to overall 2D normal tension (*σ*) within the cells following *R* = *λ*/*σ*^35,36^ that has been used to accurately predict traction forces^37^. Force balance model analysis reveals that increased contractile forces scale with increasing *R*^28^, following *R* = (**F**_M_/*σ*) × (*L*/(*L* − *L*_0_)), where **F**_M_ is the average myosin-generated stress in the actomyosin assembly cross-section and relates to the linear tension following *λ* = (**F**_M_) × (*L*/(*L* − *L*_0_)), where *L* relates to the actomyosin assembly length, and *L*_0_ is a parameter that depends on the effective friction and viscosity of actin filaments resistance to shear. Thus *R* increases linearly with myosin contractile forces that directly influence traction forces at the cell–matrix interface, particularly for our grid architecture that is specifically suited for formation of free cell edges between adhesions on two adjacent orthogonal lines, similar to adhesion on fiber networks, and consistent with reports demonstrating correlation between larger forces at adhesion sites and increased peripheral actin fibers with larger *R*^37^. In addition, the data shows a linear correlation between *R* and spanning distance, *d*^28,35,36^, thus we also calculated the *R*/*d* metric since our system allows for adhesion at different distances (i.e., different spanning distances; Fig. 1b). Furthermore, if the myosin-regulated contractility strength is too low, the system becomes unstable and arcs can collapse^28^. Our grid architecture allowed us to develop an analysis that captures the switch from elastic polygonal states to more passive non-contractile dendritic states by identifying *R*/*d* values of ~0.71 as the theoretical lower limit for any free cell edge on the orthogonal adhesive grid (Fig. 1). Thus, while active contractile cells will have higher *R* values and *R*/*d* values >0.71 and increase with larger forces at adhesion sites, low/non-contractile passive cells feature more fluidic-like mechanical behavior and a dendritic phenotype with protrusions collapsed onto the orthogonal lines, resulting in free cells edges conforming to the lines along the inscribed circle (Fig. 1d).

### The influence of ECM dimensionality and stiffness on guidance sensing

**Fig. 2:**
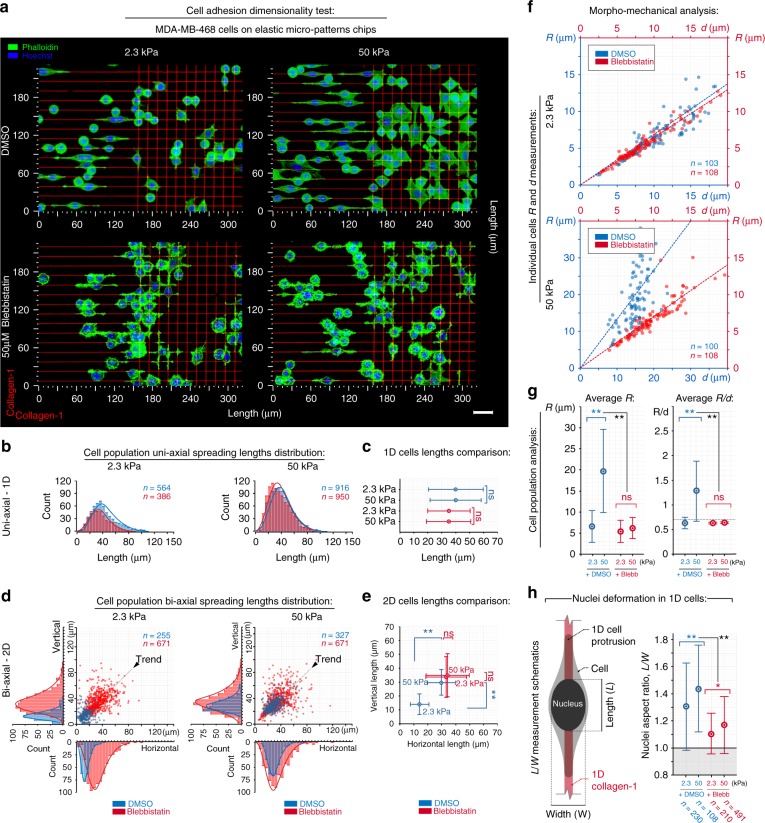
ECM dimensionality and stiffness influence guidance sensing. **a** MDA-MB-468 breast carcinoma cells sensing and protruding along uniaxial collagen lines that mimic aligned fibers and biaxial cues to mimic fiber networks (left and right halves of each micrograph, respectively; 3D reconstructions) on soft (2.3 kPa) and stiff (50 kPa) substrates under control (DMSO, Supplementary Movies 1,2,3) and blebbistatin-treated conditions. Blebbistatin-treated cells display a dendritic protrusion phenotype in response to uniaxial (Supplementary Movie 4) and biaxial (Supplementary Movie 5) cues on both soft and stiff substrates, suggesting the presence of a bimodal response to guidance cues that depends on cell traction stress magnitudes. **b**, **c** Morphometric analysis of cells on soft and stiff uniaxial guidance cues for control conditions (blue) and blebbistatin treatment (red). For both conditions, uniaxial collagen lines induce a robust guidance response with no significant differences in cell protrusion between soft and stiff substrates. All corresponding *n* values in **c** are shown in **b**. **d**, **e** Morphometric analysis of cells interacting with biaxial guidance cues in control (blue) and blebbistatin-treated conditions (red), with significantly greater protrusion on stiff versus soft substrates for control cells. Blebbistatin-treated cells display no detectable difference in their biaxial guidance response on soft and stiff substrates. All corresponding *n* values in **e** are shown on **d**. **f**, **g** Morpho-mechanical analysis of cells of soft and stiff substrates showing significantly higher values for both *R* and *R*/*d* metrics in response to stiff substrates, representative of higher cell traction forces in response to stiff substrates. In contrast, soft substrates or inhibition of contractile forces result in low *R* values and mean *R*/*d* values near the threshold for elastic cell behavior. All corresponding *n* values and individual data points in **g** are shown in **f**. **h** Nuclei deformation, where lateral compression results from cell tension alignment, as a reference for contractile force magnitude. Scale bar in **a**—15 µm. Corresponding *n* values are shown on the plots. Number of replicates (independent experiments) for all measurements *N* = 4. Data in **c**, **e**, **g**, **h** are mean ± s.d.; ns: no significant difference between groups; *p < 0.05, **p < 0.001 (ANOVA)

**Fig. 3:**
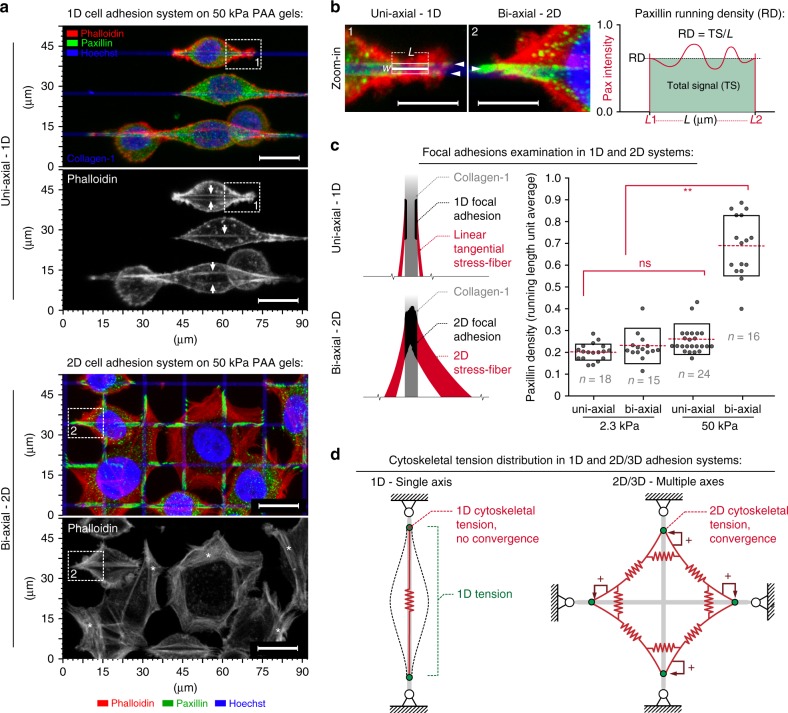
Cell adhesion and mechanical responses to distinct guidance cue dimensionality and rigidity. **a** Fluorescence 3D reconstruction micrographs of F-actin (phalloidin—red), FAs (paxillin—green), and nuclei (Hoechst—blue) on stiff uniaxial and biaxial platforms reveal a more robust development of the FA and stress-fiber contractile apparatus in response to biaxial cues versus 1D cues. White arrows (top panel) highlight actin fibers. Boxes 1 and 2 are zoomed-in regions shown in **b**. **b** Zoomed-in apical regions shown in **a**. Arrowheads highlight prominent FA development homogeneously filling the line in response to biaxial cues (2) while paxillin distribution for uniaxial cues is more localized at the line edges (1). To capture differences in FAs, we employ a metric of running length density. Paxillin running density is an “intensive” parameter that reflects the total paxillin signal per micron of length along collagen guidance cue axes to characterizes the build-up of FAs in terms of structural density, maturity, and level of integration with contractile stress-fibers (see right panel). **c** Schematic representation of the observed FA and corresponding stress-fiber architectures for cells encountering uniaxial or biaxial cues and quantification of FA running length densities for soft and stiff 1D and biaxial substrates (**p < 0.001). **d** Model of the cell mechanical response during sensing of uniaxial and biaxial cues. The uniaxial guidance cues result in highly anisotropic forces guided along adhesions and the actin fiber cytoskeleton (CSK) that are co-aligned with the uniaxial cues such that linear tension (*λ*) is distributed along the CSK structure. In contrast, biaxial cues provide opposing forces across a wide range of angles and adhesion sites resulting in robust mechanical feedback loops that promote traction stresses, FA maturation, and stress-fiber formation, inducing robust linear tension along arcs and formation of associated stress-fibers (bottom panels of **a**) that converge to generate higher stress magnitudes at FAs. Scale bars—15 µm. Corresponding *n* values are shown on the plot. Number of replicates (independent experiments) for all measurements *N* = 2. Data in **c** are mean ± s.d (boxes); ns indicates no significant difference between groups; *p < 0.05, **p < 0.001 (ANOVA)

To develop further insight into carcinoma cell contact guidance, we first determined the impact of both rigidity and ECM dimensionality (i.e., uniaxial 1D mimicking aligned fibers and biaxial 2D mimicking fiber networks). Cells encountering uniaxial cues orient robustly along collagen lines to display 1D behavior, while biaxial cues result in a polygonal phenotype with protrusions in both directions. Perhaps surprisingly, we found that changing the rigidity (2.3–50 kPa) had no effect on sensing with similar orientation and elongation observed in response to uniaxial guidance cues (Fig. 2a–c, Supplementary Movies 1 and 2). On the contrary, cells on adjacent 2D grids display strong stiffness-dependent differences in behavior, with cells on stiffer substrates developing significantly greater protrusive spreading (Fig. 2a, d, e, Supplementary Movie 3) and cell forces as demonstrated by significant increases in *R* and *R*/*d* metrics on stiff substrates (Fig. 2f, g). This mechanoresponsive behavior likely results from the biaxial nature of the system where opposing forces from mature FAs across a wide range of angles provides robust feedback to promote traction stresses, FA maturation, and stress-fiber formation (Fig. 3), consistent with findings in NIH3T3 cells where greater FA sizes and stronger traction forces are present on planar 2D surfaces versus 1D lines^39^. Indeed, in response to uniaxial cues, carcinoma cells have less developed FAs and stress-fibers compared to the response to biaxial architectures (Fig. 3). Yet, analysis of nuclear deformation, an established metric of cell contractility state where lateral compression results from cell tension alignment^41^, indicates that cells responding to stiff uniaxial cues are in a more contractile state even though guidance sensing and protrusion lengths are not different on soft versus stiff uniaxial cues (Fig. 2h), suggesting the possibility of compensatory mechanisms to maintain contact guidance in soft environments. Likewise, we note that mechanoresponsiveness is not present on flat nanoprinted substrates where 800 nm collagen lines are 800 nm apart representing a quasi-2D surface (Supplementary Figure 3a), further highlighting the need to compare uniaxial guidance cues to network configurations as opposed to planar or quasi-2D environments (fibrous versus continuous ligand architectures). Thus we conclude that carcinoma cells with aligned and anisotropic, yet not large traction forces effectively sense and respond to both soft and stiff uniaxial paths. In contrast, substrate stiffness-dependent behavior exists in response to biaxial cues, suggesting that the direction and organization of the aligned contact guidance cues are likely more dominant for guidance than matrix stiffness (Fig. 3).

To test the hypothesis that the mechanosensitive response to biaxial cues is cell traction-dependent while the response to uniaxial cues is largely traction-independent, we selectively suppressed myosin II contractility with blebbistatin. As anticipated, after diminishing contractility, cells responding to uniaxial cues show no significant difference in their ability to sense and protrude along soft and stiff collagen lines (Fig. 2a–e, Supplementary Movie 4), which may be consistent with observations on fibronectin where nascent adhesions replace stable FAs during 1D fibroblast protrusions following myosin inhibition^42^ and a recent report demonstrating the ability of blebbistatin-treated HT-1080 fibrosarcoma cells to sense fibrous fibronectin-coated guidance cues^43^. Yet, the impact of matrix mechanics in regulating bimodal behaviors and the molecular mechanisms governing guidance sensing in low and high contractility states remains largely unknown. Similar to the uniaxial condition, low-traction cells responding to biaxial cues show no significant difference in their ability to sense and protrude along soft and stiff collagen lines (Fig. 2d, e). However, on both soft and stiff substrates we observed a profound phenotypic shift toward a mode of dendritic protrusion, where carcinoma cells develop long, thin, and branched protrusions that are tightly guided by, and *flow* along, the grid architecture (Fig. 2a, Supplementary Movies 5 and 6). Indeed, with blebbistatin, *R* and *R*/*d* metric values are at or below the critical value for elastic cell behavior suggesting that they are in the more passive fluidic-like mechanical state with a dendritic phenotype. Notably, this directed behavior is distinct from previously reported blebbistatin-induced random dendritic cell protrusions in fibroblasts on continuous 2D substrates^44^, which have been reported to require intact microtubule (MT) structure to robustly form^44^. Here we observed that for carcinoma cells without robust myosin motor activity, dendritic protrusions require MTs (±nocodazole treatment) on quasi-2D surfaces but not in response to biaxial cues where they are dispensable during development of guided dendritic protrusions, as well as dendritic-directed migration (Supplementary Figure 4a, Supplementary Movie 7). Thus distinct mechanisms appear to drive contact-guided protrusion dynamics when compared to random protrusions on flat 2D surfaces. Indeed, in the absence of sufficient traction forces, our data suggest that carcinoma cells may be able to switch to an alternative mechanism of sensing and protruding along guidance cues.

### Bimodal sensing of guidance cues

Our findings that the response to 1D guidance cues is not dependent on substrate rigidity nor cell contractility suggests that carcinoma cells can deploy a high-traction-independent approach for effective ECM pattern sensing and protrusion. To test our hypothesis and define the molecular mechanisms governing contact guidance in both high- and low-traction states, we first explored the role of Arp2/3-dependent actin nucleation and formins, which are required for effective transmission of traction force between FAs and the actin cytoskeleton^24–26,45^ and have been recently reported to influence adhesion stability and 3D matrix deformation that influences invasion^26^, yet their role in contact guidance is less explored. Furthermore, both Arp2/3 and formins have been widely implicated in lamellipodia and filopodia dynamics^27,28^; however, their role during distinct protrusion and motility events is not without controversy. For example, Arp2/3 has been reported to be essential for both random and directed cell protrusion and migration in some contexts, such as haptotaxis, but dispensable in others^28,46,47^. Furthermore, we note that in fibroblasts loss of Arp2/3 complex is required for formation of random dendritic protrusions in the presence of blebbistatin^28^, distinct from our findings here that blebbistatin is sufficient for formation of guided dendritic protrusions in carcinoma cells (Fig. 2), which may be more aligned with T cell behavior, where blebbistatin does not suppress T-cell receptor-induced protrusions that are reported to be dependent on actin polymerization and indeed Arp2/3-regulated actin dynamics^48^. We therefore hypothesized that Arp2/3-dependent F-actin polymerization facilitates fluidic-like guidance dynamics through F-actin polymerization with short, branched F-actin assembling into a meshwork that mediates fine conformity of protrusions along adhesion cues, while formins are necessary for high-traction-dependent contact guidance.

**Fig. 4:**
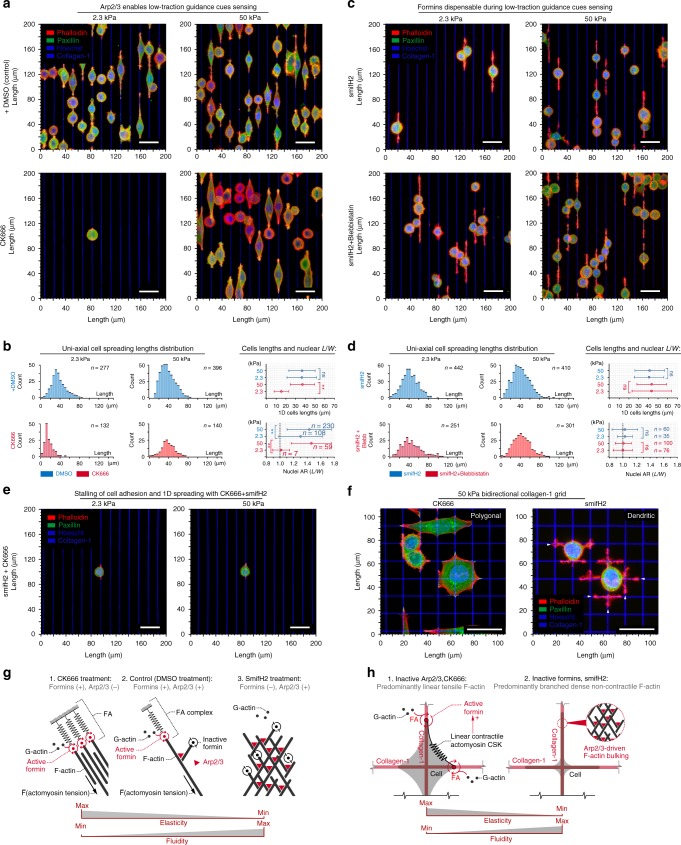
Bimodal sensing of uniaxial contact guidance cues. **a** Carcinoma cells sense soft (2.3 kPa, Supplementary Movie 1) and stiff (50 kPa, Supplementary Movie 2) uniaxial matrix (top panels; 3D reconstruction). Following Arp2/3 inhibition (bottom panels), cells do not respond to soft uniaxial cues (Supplementary Movie 8) but protrude along stiff substrates (Supplementary Movie 9; Supplementary Figure 4b). **b** Morphometric analysis of cells on soft and stiff uniaxial guidance cues in control (blue) and CK666-treated conditions (red) showing significantly reduced guidance sensing following Arp2/3 inhibition on soft substrates, where traction forces are relatively low but not on stiff matrix. Likewise, greater nuclei deformation, representative of cell contractility, is observed on stiff substrates, where no difference is observed between control and Arp2/3-inhibited cells, while Arp2/3-inhibited cells on soft substrates show no nuclei deformation (AR ~ 1). **c** Cells sense soft and stiff uniaxial matrix under formin inhibition (top panels) using the low-traction dendritic protrusion phenotype (3D reconstruction; confirmed by adding blebbistatin—bottom panels). **d** Morphometric analysis of cells on soft and stiff uniaxial cues with smifH2 (blue) or smifH2+blebbistatin (red). Lack of nuclei deformation (AR ~ 1) highlights the loss of traction from disruption of the force transmission linkage (formins) or motor activity (blebbistatin). **e** Simultaneous inhibition of the formin-dependent traction-driven and Arp2/3-dependent traction-independent modes of guidance sensing completely abrogates the cell contact guidance response. **f** Carcinoma cells respond to stiff biaxial cues following Arp2/3 inhibition (Supplementary Movie 12) but switch to the low-traction dendritic protrusion phenotype (white arrowheads) under formin inhibition (Supplementary Movies 10 and 11). **g** Modalities of F-actin polymerization and cell protrusion. Abrogation of formins leads to solely Arp2/3-driven F-actin polymerization (branching), resulting in dense F-actin bulking that conforms to guidance cues (more fluidic-like behavior). Suppression of Arp2/3 shifts cells to formin-dependent behavior with linear F-actin transmitting forces (elastic dynamics). **h** Schematics for polygonal and dendritic spreading dynamics controlled by formins and Arp2/3. Scale bars—30 µm. Corresponding *n* values are shown on the plots. Number of replicates (independent experiments) for all measurements *N* = 4. Data in **b**, **d** are mean ± s.d., ns: no significant difference between groups, *p < 0.05, **p < 0.001 (ANOVA)

We first tested our hypothesis by quantifying the response to soft and stiff 1D architectures in the presence and absence of Arp2/3 activity. Examining cells in the lower-traction state resulting from soft 1D substrates allowed us to directly test Arp2/3 dependence without needing to suppress cell contractility. We hypothesized that Arp2/3-driven protrusion can compensate for loss of high-traction-dependent cell protrusion dynamics and thus predicted that suppression of Arp2/3 should inhibit uniaxial guidance on soft but not stiff substrates. Indeed, as predicted, Arp2/3 inhibition (+CK666) suppresses the response to uniaxial guidance cues on soft but not stiff substrates and also results in smaller FAs that are tightly localized at the periphery of the cell–collagen interface (Fig. 4a, c, f, Supplementary Figures 4b, 6a, Supplementary Movies 8, 9). These results were independently confirmed with a second Arp2/3 inhibitor (+CK869) with a distinct mechanism of action (Supplementary Figure 4b) and identify a matrix stiffness-dependent role for Arp2/3 in contact guidance, which is distinct from work suggesting that a cell autonomous role for Arp2/3 is necessary for chemotactic-directed migration^47^. In addition, Arp2/3 suppression transformed cell protrusion dynamics from oscillatory advancement–retraction cycles toward linear steady advancement (Supplementary Figure 5). Thus the fact that cells are capable of recognizing uniaxial cues on stiff ECM without Arp2/3 activity highlights the presence of two coexisting yet distinct mechanisms for guidance sensing. Namely, a mechanosensitive traction stress feedback system and an Arp2/3-dependent mechanism during low-traction states that promotes a more fluidic-like cell elongation phenotype.

To further explore these conclusions, we sought to identify a role for formins in sensing of guidance cues by suppression of the formin superfamily with smifH2. Disruption of the mechanical link between the actin cytoskeleton and ECM via formin inhibition does not prevent guidance sensing (Fig. 4c, d, Supplementary Figure 6b). Strikingly, suppression of formins reduces FAs and shifts cells to the low-traction protrusion phenotype along both soft and stiff uniaxial lines, with thin protrusions along guidance cues and loss of nuclear aspect ratio (Fig. 4c, d), replicating blebbistatin treatment results (Fig. 2). To verify the smifH2-induced low-traction contact guidance phenotype, we combined smifH2 with blebbistatin treatment and observed no change from the addition of blebbistatin (Fig. 4c, d). We next tested whether the formin inhibition-induced protrusion phenotype relies on Arp2/3-driven F-actin dynamics. Indeed, consistent with our hypotheses and conclusions, inhibition of both formins and Arp2/3 completely abrogates the ability of carcinoma cells to sense contact guidance cues (Fig. 4e), and also greatly reduces cell adhesion and protrusion on both soft and stiff guidance cues. This is further supported by examination of the response to biaxial cues where Arp2/3-inhibited cells can still respond to stiff substrates while formins inhibition mimics the low-traction phenotype found with blebbistatin treatment (Fig. 4f, Supplementary Figure 7, Supplementary Movies 10, 11), distinct from behavior on quasi-2D flat nanopatterns (Supplementary Figure 3). Thus bimodal sensing of guidance cues is dependent on Arp2/3 or formins on soft and stiff guidance cues, respectively (Fig. 4g), where a mechanosensitive traction stress feedback system is dominant on stiff environments while the Arp2/3-dependent mechanism during low-traction states promotes a more dendritic cell elongation phenotype (Fig. 4h).

### Arp2/3 complex inhibition promotes a uniaxial response to stiff biaxial guidance cues

**Fig. 5:**
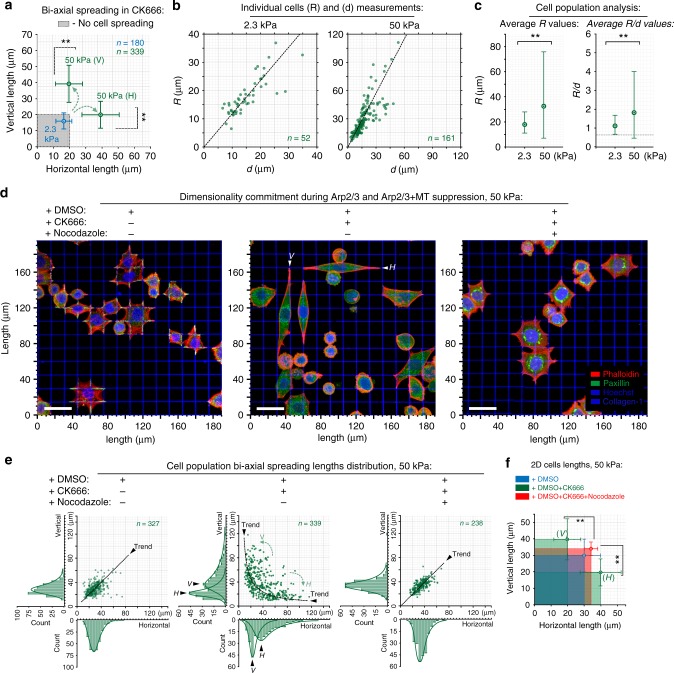
Arp2/3 suppression promotes a microtubule-dependent uniaxial guidance response. **a** MDA-MB-468 breast carcinoma cells under Arp2/3 complex inhibition (+CK666) sense soft (2.3 kPa) and stiff (50 kPa, Supplementary Movie 12) biaxial collagen guidance cues. Note that cell populations on stiff substrates split into vertical and horizontal cell subpopulations (V vertical cells, H horizontal cells). **b** Morpho-mechanical analysis of cells populations under Arp2/3 inhibition conditions on soft and stiff biaxial cues: free cell edge curvature radii, *R*. **c** Average values of *R* and *R*/*d* in cell under Arp2/3 complex inhibition, showing increased traction on stiffer substrates (see Fig. 2g for comparison). The corresponding *n* values are shown on **b**. **d**, **e** 3D reconstruction overviews and morphometric analysis of the Arp2/3-inhibited cell population response to biaxial guidance cues. Inactivation of Arp2/3 (+CK666) induces non-equidirectional cell protrusion with random cell commitment to either of the two orthogonal directions (i.e., a uniaxial response). Disruption of intact microtubules in cells with suppressed Arp2/3 complex (+ CK666+nocodazole) rescues biaxial cell spreading. **f** Average values of cell lengths from protrusion for each of the conditions in **d**, **e** (see color code). The corresponding *n* values are shown on **e**. Scale bars—30 µm. All corresponding *n* values are shown on the plots. Number of replicates (independent experiments) for all measurements *N* = 4. Data in **a**, **c**, **f** are mean ± s.d.; ns: no significant difference between groups; *p < 0.05, **p < 0.001 (**c**: unpaired *t* test; **a**, **f**: ANOVA)

Arp2/3 inhibition unmasks a steady advancement of protrusions along stiff collagen guidance cues, whereas in non-contractile cells, Arp2/3 is necessary for protrusion along guidance cues (Fig. 4, Supplementary Figures 5–7). Furthermore, while cells respond to stiff biaxial substrates with and without Arp2/3 complex activity (Figs. 2 and 4), inspection of Arp2/3-inhibited cells suggests large subpopulations of cells oriented along either the horizontal or vertical axis but not both (Fig. 4f). Thus we hypothesized that, in contractile cells, Arp2/3 activity may also promote multiaxial protrusion dynamics in concert with tension-driven FA dynamics (Fig. 5). Indeed, quantitative evaluation of cell behavior on soft and stiff biaxial cues under Arp2/3-complex inhibition reveals a minimal response to soft cues (Fig. 5a–c), as expected, but a distinct response on stiff substrates, where cells predominantly commit to either horizontal or vertical guidance cues (Fig. 5a, Supplementary Movie 12), in contrast to the more uniform response under control conditions (Fig. 2a, d, e). With equal probability, cells appear to spontaneously linearize and acquire a uniaxial response phenotype (Fig. 5a), while retaining increased traction behavior on soft versus stiff substrates (Fig. 5b, c). Thus our data suggest that on stiff environments Arp2/3-complex signaling is necessary for carcinoma cells to robustly interact simultaneously with differentially aligned biaxial fibrous network guidance cues.

**Fig. 6:**
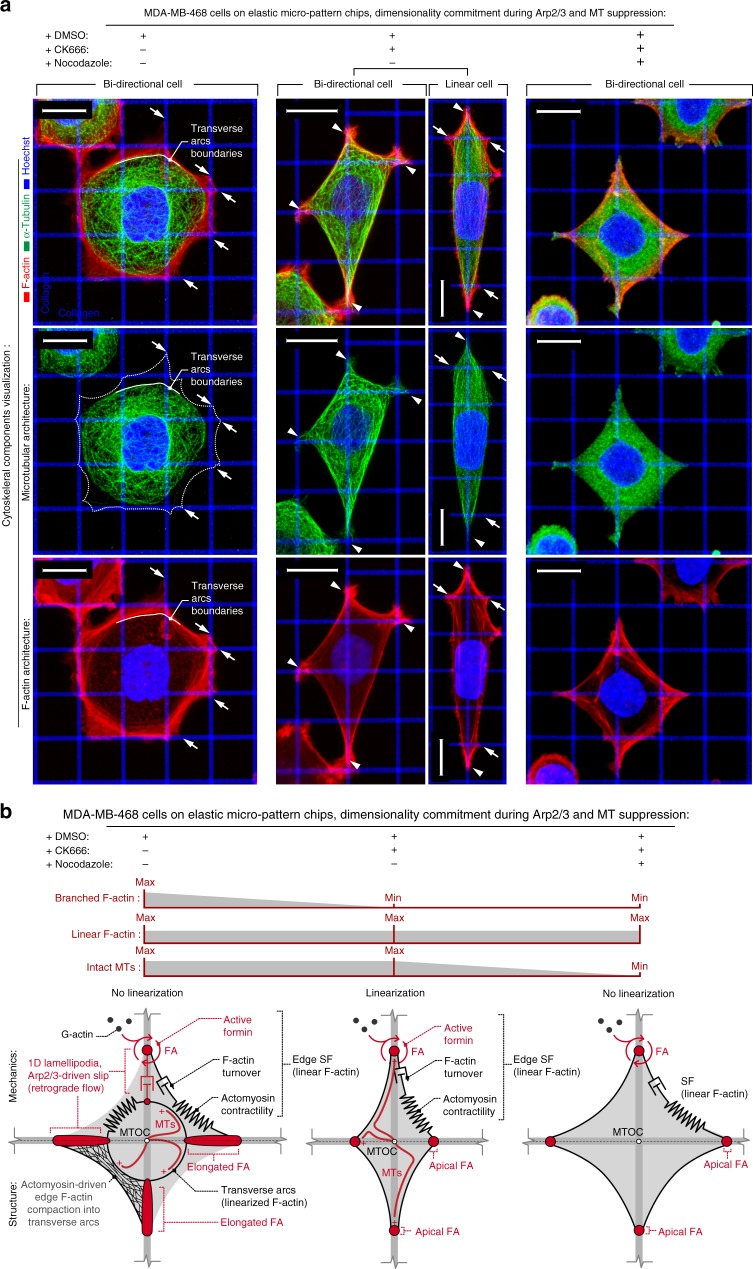
Arp2/3 suppression-induced cytoskeletal and cell architecture reorganization. **a** 3D reconstructions of MDA-MB-468 cells in control (+DMSO), under Arp2/3 inhibition (+CK666), and Arp2/3 inhibition without intact microtubules (+nocodazole+CK666). Control cells develop peripheral stress-fibers and also transverse arcs (see also Figs. 1c and 3a) that spatially coincide with boundaries of microtubular network, preventing microtubule localization at the cell spreading apices (white arrows), whereas Arp2/3 inhibition shifts the balance toward peripheral stress-fibers and formation of concentrated FAs at the cell periphery at points of the cell–matrix interface (see also Fig. 4f and Supplementary Figure 6). Furthermore, in Arp2/3-abrogated cells, the microtubule network conforms to the boundaries to the peripheral stress-fibers and cell apices (white arrowheads). In contrast, no intact microtubule networks are observed after nocodazole treatment. **b** Schematic representation of actomyosin and microtubular cytoskeleton components in control cells and in cells under Arp2/3 (±intact MTs) inhibition conditions. Note actomyosin contractility along the peripheral stress-fibers and its compaction along the transverse arcs where the transverse arcs boundaries prevent microtubule localization to the cell adhesion boundaries in control cells. Arp2/3 inhibition causes suppression of transverse arcs, allowing the microtubule network to connect to the cell adhesion boundaries, resulting in more isolated peripheral stress-fibers and diminished 1D lamellipodia slip. Removal of intact MT network rescues cells from CK666-mediated uniaxial linearization. Scale bars—15 µm

Intact MTs were required for the randomly directed dendritic (i.e., multidirectional) mode of cell protrusions on quasi-2D substrates (dense nanolines) but were dispensable for bidirectional dendritic cell spreading guided along biaxial collagen grids (Supplementary Figure 4a). We therefore hypothesized that Arp2/3-activity-related uniaxial response to biaxial guidance cues is dependent on MTs. Consistent with our hypothesis, disruption of MTs (+nocodazole) in Arp2/3-inhibited cells results in complete loss of uniaxial commitment (Fig. 5d–f), suggesting a structural and mechanical relationship between linearized actin fiber and MT networks during commitment of a uniaxial contact guidance response^49,50^. Examination of F-actin and MT cytoskeletons in control cells demonstrates that during sensing of biaxial guidance cues actin fibers and MTs are not co-localized (Fig. 6a). In this case, F-actin is found in transverse arcs and peripheral stress-fibers (Fig. 6a, b), where Arp2/3-facilitated 1D lamellipodium formed on the collagen lines fuses peripheral stress-fibers together and drives their dynamic slip into F-actin-compacting transverse arcs rings, detected within the boundaries of elongated FAs (Fig. 6b, Supplementary Figure 6). This is a well-established phenomenon reported for various cells on continuous substrates, where this process facilitates reorientation and transitioning between cell-adhesion-aligned radial dorsal/ventral stress-fibers, and a non-oriented network of transverse arcs^51^, which may have a significant implication during cell sensing and alignment toward oriented collagen lines in bidirectional grids. We find that transverse arcs of linear F-actin appear to trap the majority of MTs and prevent them from interacting with peripheral stress-fibers and therefore restrict interactions with the portion of actomyosin network that is localized near cell adhesions (Fig. 6a, b). Indeed, various reports indicate the localization of conventional MTs–F-actin adaptor septin network at the edge between transverse arcs and radial stress-fibers^49,52^. In contrast, during uniaxial commitment resulting from Arp2/3 inhibition, F-actin morphology shifts from transverse arcs dynamics toward peripheral stress-fibers where MTs now directly interact with peripheral stress-fibers at the cell boundaries and adhesion sites, raising interesting questions as to whether cell linearization during the uniaxial response results from MT–actomyosin bundling (Fig. 6a, b). Removal of MTs with nocodazole prevents cell linearization, even though linear F-actin predominantly remains as peripheral stress-fibers (Fig. 6a, b and Supplementary Figure 6a). Combined, this suggests a model where a well-established multitude of interactions between MTs and contracting peripheral F-actin stress-fibers (via conventionally known MT–actomyosin interactions adaptors, such as myosin, and TIP^50^ and septin^49^ complex, which can be overexpressed in some carcinoma cells^52^) are necessary to promote Arp2/3-dependent uniaxial dimensionality commitment on biaxial contact guidance cues (Fig. 6b).

### Guidance and stiffness sensing during combined cell–cell and cell–ECM interactions

We previously demonstrated that forces across cell–cell interactions can compete with forces from integrin–ECM interactions to diminish the contact guidance response^11^. To evaluate the impact of forces across cell–cell adhesions during guidance sensing, we further advanced our biaxial platform to include concurrent cell adhesion along two normally oriented adhesion ligands (i.e., collagen–cadherin for competing cell–ECM versus cell–cell interactions; Supplementary Figure 8). This sensing-and-protrusion matrix facilitates high spatial segregation of adhesion ligands but retains biomechanical coupling between the two systems^48^. Perpendicular lines on substrates of varying stiffness allow cells to encounter mimetic low and high normally oriented forces across E-cadherins, which is the direction of forces across E-cadherin during cell–cell interactions^18^, an organization that is distinct from previous substrates that patterned basement membrane collagen (type IV) and E-cadherin as alternating adjacent parallel stripes^16^. We confirmed the specificity of the collagen and E-cadherin ligands through immunofluorescence staining, where no cross-reactivity was observed (Supplementary Figure 9). Moreover, we confirmed that no ECM ligand had absorbed to the E-cadherin lines by observing the response of MDA-MB-231 breast carcinoma cells that lack expression of E-cadherin^11^. As expected, MDA-MB-231 show no response to the E-cadherin lines confirming that no ECM is present to produce protrusion from integrin-mediated adhesions (Supplementary Figure 9).

**Fig. 7:**
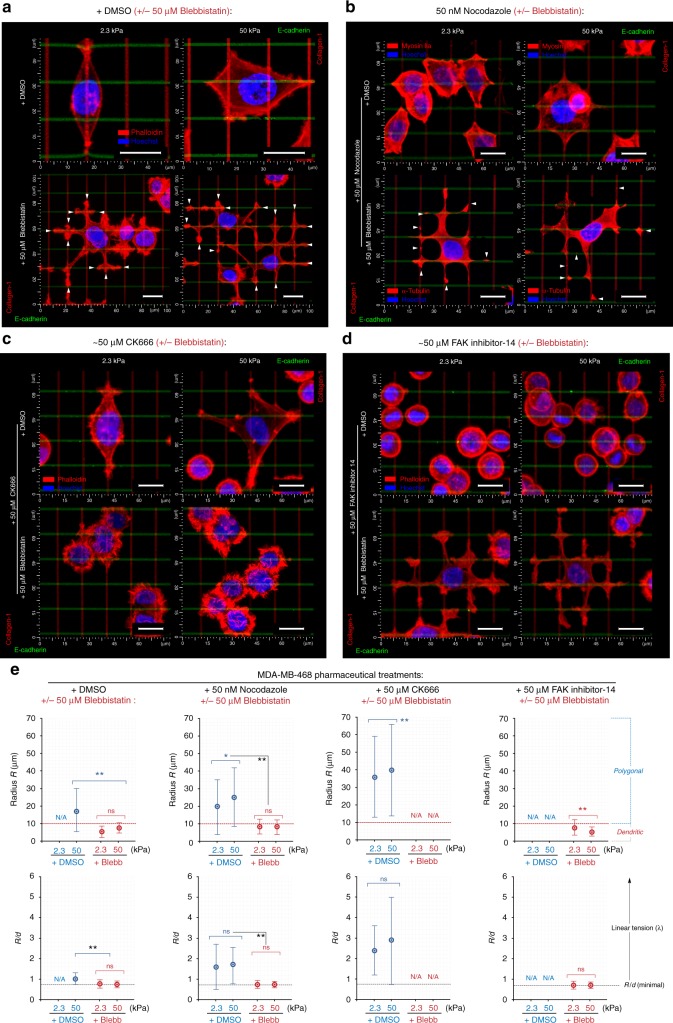
Guidance and stiffness sensing on competing cell–ECM and cell–cell interactions. **a**–**d** 3D reconstructions of MDA-MB-468 cells spreading on the collagen (vertical, red) versus E-cadherin (horizontal, green) orthogonal elastic grids. **a** MDA-MB-468 breast carcinoma cell sensing and protruding along competing collagen and E-cadherin cues on soft (2.3 kPa) and stiff (50 kPa) substrates under control conditions and in the presence of blebbistatin, where the dendritic protrusion phenotype emerges in response to both collagen and E-cadherin. Note that cells on soft substrates do not respond to E-cadherin cues. **b**–**d** Breast carcinoma cell response to collagen and E-cadherin cues on soft and stiff substrates in response to **b** microtubule disruption (nocodazole), **c** Arp2/3 inhibition, or **d** FAK inhibition, with ±blebbistatin treatment for each condition. Following microtubule disruption, cells are no longer biased along collagen on soft substrates but instead respond to both collagen and E-cadherin ligands on both soft and stiff substrates. Inhibition of Arp2/3 complex does not substantially impact contractile cells but ablates low-traction-dependent dendritic protrusions along both collagen and E-cadherin while inhibition of FAK disrupts the guidance sensing response in contractile cells but is dispensable for Arp2/3-dependent dendritic protrusion. **e** Morpho-mechanical analysis of cells on soft and stiff substrates for each experimental condition. Note, we define cells with *R* > 10 µm as polygonal and cells with *R* < 10 µm as dendritic, consistent with the lower limit for *R*/*d* as ~0.71. The corresponding *n* values are shown in Supplementary Figure 10a. Scale bars—15 µm. Number of replicates (independent experiments) for all measurements *N* = 5. Data in **e** are mean ± s.d.; ns: no significant difference between groups; *p < 0.05, **p < 0.001 (ANOVA). See also Supplementary Figure 10a for the raw data of population-wide distribution of individual *R* and *d* measurements

**Fig. 8:**
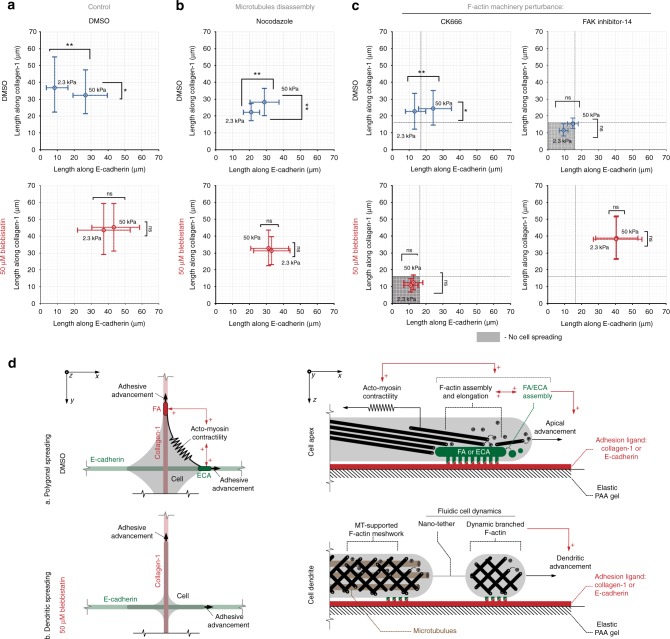
Physical and molecular mechanisms governing bimodal sensing of contact guidance cues in carcinoma cells. **a**–**c** Summary of the bimodal cellular guidance sensing response for **a** control conditions, **b** disruption of microtubules, and **c** Arp2/3 and FAK inhibition, respectively, demonstrating a bimodal response for sensing guidance cues that depends on the magnitude of cell traction forces and two parallel contractile force-dependent mechanisms where FAK regulates the response in contractile cells and Arp2/3 regulates sensing in the low-traction state. The corresponding *n* values for **a**–**c** are shown in Supplementary Figure 10b. **d** Model of the carcinoma cell-sensing response in the contractile elastic state and the more fluidic-like, low-traction dendritic state where protrusive forces from branched actin network dynamics drive adhesion-directed sensing of guidance cues. Number of replicates (independent experiments) for all measurement *N* = 5. Data are mean ± s.d., ns: no significant difference between groups, *p < 0.05, **p < 0.001 (ANOVA). See also Supplementary Figure 10b for the raw data of population-wide individual cell spreading length measurements compiled into distribution diagrams

Combined E-cadherin and collagen grids induce competitive guidance dynamics (Figs. 7 and 8). Cells on stiff substrates display a polygonal phenotype resulting from the tug-of-war mechanical competition between the two simultaneously engaged and spatially separated adhesion systems that each bear cell tractions (Fig. 7a, e). In contrast, cells on soft substrates only orient and protrude along collagen lines (Figs. 7a and 8a) suggesting that mechanical competition exists between E-cadherin and integrin adhesion subsystems and that cadherin may require relatively large forces to compete with forces across cell–matrix adhesions. To confirm that this competition would break down in the absence of traction forces, we treated cells with blebbistatin. Surprisingly, carcinoma cells developed dendritic protrusion phenotypes along both collagen and E-cadherin in the absence of significant traction forces (Fig. 7a, e) as noted by *R*/*d* values near or below the theoretical limit (Fig. 7e), indicative of low/non-contractile passive cells displaying a dendritic phenotype. Thus the carcinoma cells evaluated here are able to produce contact-guided protrusions along both collagen and E-cadherin ligands under both low- and high-cell-traction conditions. While the full implications of this behavior are not currently known, it does suggest that carcinoma cells may be capable of sampling larger areas of cell–cell ligand interactions (i.e., interactions with single or multiple cells at multiple cell lengths or protrusions connecting to multiple cells) than previously appreciated.

In order to dissect the physical and molecular mechanisms governing the distinct traction-dependent and -independent behaviors observed here and further define the role of cytoskeletal dynamics in regulating guidance sensing, we perturbed a panel of key elements that we hypothesized, based on our findings in response to collagen guidance cues, to be regulatory elements. Namely, we tested the hypothesis that MT stability, Arp2/3-complex, and FAK signaling each play a central role in contact guidance. Following disruption of MTs with nocodazole, cells are no longer biased along collagen on soft substrates, rather, they protrude along both collagen and E-cadherin ligands on both soft and stiff substrates (Figs. 7b, e and 8b). Yet, protrusion developed in a mechanoresponsive manner (Figs. 7b and 8b), which is consistent with reports of higher cell contractility following MT disassembly^53^ and our observation for higher *R* and *R*/*d* metrics (Fig. 7e, Supplementary Figure 10), which may result in increased traction forces that appear necessary to robustly activate the E-cadherin system. Perhaps surprisingly, but consistent with findings in response to biaxial collagen guidance cues (Supplementary Figure 4a), MT disruption does not suppress the formation of blebbistatin-induced dendritic protrusions on soft or stiff substrates (Figs. 7b, e and 8b, Supplementary Movie 7). Cells display an extreme morphological conformity to the guidance cues (*R*/*d* values near ~0.71) and often develop micro-fragments that split from the cell body and appear to be attached only via nanoscale tethers (Fig. 7b). Interestingly, MT-positive cells do not display fragmentation (Fig. 7a), suggesting that, for cells in a low-traction state, MTs may provide structural integrity to constrain more passive protrusion dynamics.

To test whether guidance sensing with dendritic protrusions is Arp2/3 dependent in response to E-cadherin, as well as collagen (Fig. 4), we disrupted Arp2/3 activity in both contractile and blebbistatin-treated cells on both soft and stiff substrates (Figs. 7c and 8). Following Arp2/3 inhibition, contractile cells on soft substrates continue to orient along collagen lines, as well as demonstrate mechanosensitive behavior, albeit with protrusion lengths that are somewhat diminished (Figs. 7a, c, e and 8c). However, the uniaxial response to biaxial cues observed for collagen cues is not present in the combined E-cadherin–collagen system (Supplementary Figure 10b), suggesting some distinct function of Arp2/3 dynamics between cell–cell and cell–ECM interactions. In stark contrast to behavior in contractile cells, Arp2/3 inhibition concomitant with contractility inhibition results in a complete abrogation of guidance sensing (Figs. 7c and 8c), consistent with findings following combined Arp2/3 and formin inhibition (Fig. 4) and an inability to perform morpho-mechanical analysis due to cells retaining a rounded phenotype (Figs. 7c, e and 8c). Hence, these data support our conclusions for a bimodal guidance sensing mechanism with a switch in fundamental protrusion dynamics depending on the level of contractile forces and, in particular, transmission of forces between the cell and its environment, for both E-cadherin interactions and cell–ECM interactions.

Inhibition of FAK phosphorylation, which is a robust regulator of FA dynamics during cell motility and known to regulate the response to mechanical signals^23,54,55^, effectively abrogated protrusion of the contractile cells along both collagen (i.e., cell maintained a rounded phenotype) and, perhaps surprisingly, E-cadherin ligands (Fig. 7d) on both soft and stiff substrates (Fig. 8c). Further, no significant differences were observed in the cellular response to soft and stiff substrates (Figs. 7d, e and 8c), consistent with reports that FAK is necessary for mechanosensing^40,54^. Strikingly, FAK inhibition in non-contractile cells does not prevent the formation of dendritic protrusions, irrespective of the underlying substrate rigidity (Figs. 7d and 8c, Supplementary Figure 10), supporting our hypothesis of two distinct mechanically regulated mechanisms governing protrusion dynamics during guidance sensing. Indeed, the data presented here, and particularly comparison of contractile and non-contractile cells with FAK or Arp2/3 inhibition, unambiguously reveals two distinct alternative guidance sensing cell protrusion mechanisms (Fig. 8c, d). While orientation and protrusion dynamics during guidance sensing is strongly formin- and FAK-dependent in contractile cells, Arp2/3 is largely dispensable (Figs. 4 and 8). Conversely, dendritic protrusions from non-contractile cells displaying more fluidic-like behavior are strongly dependent on Arp2/3, but not meaningfully impacted by FAK inhibition (Figs. 4 and 8), suggesting that Arp2/3-driven, low-traction cell protrusion only requires minimal adhesion for plasma membrane stabilization and directionality during contact guidance sensing. Thus we suggest a model for guidance sensing in cells with robust contractility that is regulated by force transmission along the integrin-acto-myosin force linkage, which can be replaced with an alternate actin cytoskeleton-driven protrusion mechanisms during guidance sensing in low-traction conditions (Fig. 8d).

## Discussion

The work here represents a focused investigation of the physical and molecular mechanisms regulating contact guidance sensing, and in particular, the impact of cell-intrinsic traction versus traction signaling feedback regulated by ECM mechanics, an exploration that has been limited in the existing literature. To perform these studies, we generated platforms and utilized the triple-negative MDA-MB-468 breast carcinoma line model system due to its phenotypic plasticity within the EMT spectrum that allowed us to capture behavior during malignant EMT transitioning^56^. By disrupting cell contractility, we demonstrate a phenotypic switch to Arp2/3-dependent dendritic protrusion along collagen, where carcinoma cells sense guidance cues and extend long protrusions along the ligand architecture independent of matrix modulus. Interestingly, consistent with our findings, Arp2/3 activity has been reported to influence a variety of cell-dendritic-like projections, enriched with dense, short, and highly branched F-actin, such as CD4 cell projections, invadosomes, invadopodia, and Listeria comet tails^48,57–59^, suggesting that these systems may employ similar mechanisms to develop low-traction protrusions. Furthermore, our results are consistent with studies demonstrating diminished myosin motor engagement force when encountering branched actin networks^60^. Thus we suggest that loss of contractility and sensitivity to Arp2/3 inhibition are consistent with a branched F-actin network capable of generating considerable adhesion-directed pushing forces, similar to comet-tail propulsion^58,59,61^, endocytosis^62^, and T cell immune synapse formation^48,63^. Thus Arp2/3 appears to be a central regulatory element in the low-traction protrusion response during contact guidance sensing and therefore simultaneously targeting both sides of the bimodal responses may provide a rational strategy for disrupting metastatic behavior.

Various models have been suggested to describe the mechanisms governing sensing of fibrous guidance cues, such as: (1) *1D* lamellipodia constrainment that concentrates cell advancement along fibrous guidance cues patterned on glass substrates in a myosin-contractility-dependent manner^14,31^, (2) 1D cell protrusion along fibers via propulsive branched F-actin polymerization waves^64^, and (3) MT-driven dendritic protrusions along collagen fibers that emerge in low-contractility states^44^. By describing co-existing and bimodal mechanisms that depend on cell and matrix mechanics, our work provides consensus and integrates and contextualizes these mechanisms. Importantly, we show that cooperation between all three mechanisms establishes the cell’s ability to sense uniaxial and biaxial fibrous collagen cues universally across various mechanical environments. Namely, our findings integrate contradicting observations of cell mechanobiology during sensing of 1D and 2D environments^15,31,33,39^ into a single model and established that both bimodal, formin-dependent and Arp2/3-dependent, mechanisms exist concurrently and often compensate for each other upon transition between compliant and stiff microenvironments. Which also appears consistent with current physics-based models such as the motor-clutch framework^65^, which suggest that polymerization could advance protrusions during very low motor activity, consistent with our experimental observations of actin-driven protrusion in low-traction conditions^65^ Indeed, in our model, the dynamics of 1D protrusion are coherent with the contractile apparatus and allows superposition of the mechanically tuned motor-clutch system and the Arp2/3-driven fluidic-like protrusion system to provide wide mechanical and structural plasticity and untangles contradictory paradigms of cell sensing in complex fibrous microenvironments. Furthermore, we establish that contractile cells sporadically align uniaxially to stiff biaxial ECM networks upon Arp2/3 depletion. These observations shed light onto the phenomenon of cell–ECM dimensionality sensing and define the key molecular players during directed and non-directed cell–ECM accommodation. Thus, by examining cell guidance sensing in defined engineered mechanical microenvironments, the data here help clarify the mechanisms available to cells as they sense and respond to complex fibrous guidance cues.

Our observations during concurrent E-cadherin and integrin signaling complement earlier work suggesting that cells more readily form cell–cell interactions on soft substrates while they migrate away from each other on stiffer substrates^66^. In contrast, cell interactions with fibronectin-coated beads strengthened E-cadherin cell–cell adhesion, suggesting synergy between the two adhesion subsystems^67^. Yet, in both experimental settings, the mechanics of the cell–cell adhesion was not controlled. Here our platform enabled us to directly evaluate concurrent mechanical competition of cell–cell and cell–ECM adhesions. We demonstrate that rigidity >2.3 kPa is required to robustly engage E-cadherin adhesion to mechanically compete with cell–ECM interactions. Thus our work addresses open questions regarding the role of force transmission and regulation of adherens junctions as earlier work raised the possibility that the actin cytoskeleton is not engaged with E-cadherin in a manner that facilitates force generation^16^. Furthermore, by specifically providing E-cadherin ligands perpendicular to collagen, as opposed to parallel lines^16^, we could directly evaluate competition for motility, as cadherin-mediated cell–cell interactions induce strong normal forces that are disrupted in the perpendicular shear direction^18^, which is the direction of collagen alignment in our platform. Lastly, perhaps surprisingly, our data demonstrate that inhibition of FAK phosphorylation not only abrogates sensing and guidance along collagen but also disrupts sensing of E-cadherin ligand. While FAK has been reported to regulate cadherin behavior in carcinoma cells^23,68^, the mechanisms driving this behavior are not well understood. Data here raise the possibility that, in addition to a potential direct signaling role for FAK in cell–cell interactions or signal transduction crosstalk between cell–matrix and cell–cell adhesions, activation of the contractile machinery through cell–matrix interactions may be required for carcinoma cells to produce robust traction in other cell sub-systems, such as force across cell–cell junctions.

Collectively, the data here shine light on mechanisms governing sensing of aligned matrices, which direct breast carcinoma cell invasion^7–10,34^. Early studies demonstrated the relationship between aligned stromal collagen and carcinoma invasion^7,10,69^ and showed that cell-mediated collagen reorganization can promote carcinoma cell invasion^9,70^. Yet, when collagen is pre-aligned, invasion can proceed in the absence of Rho/ROCK-mediated contractile forces^9^, raising a series of questions regarding the role of intracellular forces during contact guidance sensing. Likewise, mammary tissue with increased stiffness due to matrix crosslinking or elevated mammographic density shows increased matrix remodeling and collagen alignment^8,54,71,72^ leading to increased metastasis^8^, raising a series of questions regarding the role of matrix stiffness (where collagen fibers in tumors have a range of moduli according to their strain-stiffening behavior that can be deduced from the degree of crimp), and associated mechano(signal)transduction known to promote an invasive phenotype^54^, during contact guidance. Here we reconcile a number of these questions and show that matrix alignment is likely more important than stiffness for cell orientation along guidance cues, although stiffness of contact guidance cues, or perhaps durotactic gradients, may play a yet undetermined role in migration speed and/or persistence. Furthermore, we define alternate mechanisms that breast carcinoma cells in distinct contractile states can utilize to sense both soft and stiff guidance cues and demonstrate for the first time how to robustly disrupt the contact guidance sensing response with dual treatment targeting the bimodal mechanisms facilitating sensing of contact guidance cues in distant mechanical conditions.

## Methods

### Elastic micropattern preparation

For elastic hydrogel micropatterning and nanopatterning, we adapted an intermediate substrate technique in which biotin-conjugated protein micropatterns were initially printed on coverglass, then transferred onto hydrogels by cross-linking their biotin tags to streptavidin-conjugated PAA^73^. For high-precision microcontact and nanocontact printing on the intermediate coverglass, we prepared and utilized composite elastomeric stamps^48^. Briefly, a thin layer of non-collapsing hard polydimethylsiloxane (hPDMS^74^; *L* ≤ 0.5 mm by spin-coating) was cast on photo-etched 1-μm photoresist or e-beamed poly(methyl methacrylate) masters to replicate their micropattern bas-relief. Briefly, hPDMS was prepared as a mixture of 3.4 g of VDT-731 (Gelest, Inc., Cat#VDT-731), 18 µL of Pt catalyst (Platinum(0)−2,4,6,8-tetramethyl-2,4,6,8-tetravinylcyclotetrasiloxane complex solution, Sigma-Aldrich, Cat#479543), and one drop of cross-linking modulator (2,4,6,8-tetramethyl-2,4,6,8-tetravinylcyclotetrasiloxane, Sigma-Aldrich, Cat#396281). Freshly prepared hPDMS mixture was thoroughly mixed with 1 g of HMS-301 (Gelest, Inc., Cat#HMS-301) for 30 s on vortex mixer immediately before use^74^. Then, promptly after the mixture is premixed, a thin, submicron-thick (*h* < 1 mm) layer of hPDMS was spincoated prior to its polymerization on the silicone molding matrix and baked for 30 min at 60–70 °C. Regular PDMS (1:5 curing agent/base ratio, Sylgard-184, Dow Corning, Cat#4019862) was poured atop of cured hPDMS as thick cushioning supporting layer (*h* ~ 7 mm), cured at 70 °C for 1 h, and peeled together with hPDMS, then cut into the 8 × 8 mm^2^ stamps^48,74^. Using composite stamps, we created type-I collagen micropatterns on PAA as follows: First, we microprinted or nanoprinted α-collagen-1 rabbit pAb (AbCam, Cambridge, UK, Cat#ab34710; RRID:AB_731684), conjugated with biotin, ((+)-biotin *N*-hydroxysuccinimide ester, Sigma-Aldrich, Cat#H1759; as specified by the commercial protocol) and a fluorescent tag (Alexa Fluor® succinimidyl esters, Invitrogen™, Molecular Probes®, Cat#A20000, Cat#A20003; as per commercial protocol) on a clean, prebaked intermediate coverglass (FisherFinest™ Premium Cover Glass; #1, Cat#12-548-5 P; 450 °C, baked overnight). Microstamps were coated with printed antibody at a concentration of 0.2 mg/mL in phosphate-buffered saline (PBS) by incubation for 40 min at 37 °C in a humid chamber. Stamps were then gently rinsed in deionized water and dried under a jet of air or nitrogen immediately before use. Second, glass-bottom 35 mm Petri dishes (MatTek Corp., Ashland, MA, Cat#P35G-1.0-20-C) were activated with 3-(trimethoxysilyl)propyl methacrylate (Sigma-Aldrich, Cat#6514) for covalent crosslinking with PAA gels. Third, 5 μL of PAA premixes with 5% streptavidin–acrylamide (ThermoFisher, Cat#S21379) of the defined rigidity^32^ were promptly sandwiched between the activated dish and the micropatterned coverglass immediately after adding curing catalyst (aminopropyltriethoxysilane (APS)). Briefly, in order to finely control PAA mechanical rigidity, we modulated concentrations for both 40% acrylamide (40% AA) base (BioRad, Cat#161–0140) and its cross-linking molecular chain, 2% bis-AA (BioRad, Cat#161–0142) as described elsewhere^32^. Additionally, streptavidin–acrylamide (Thermo Fisher, Cat#S21379) was added to the final concentration of 0.133 mg/mL to enable PAA gels cross-linking with biotinylated proteins of interest. Briefly, for preparation of 50 µL of *G*’ = 2.3 and 50 kPa PAA gel premixes, respectively, the following components were mixed: 40% AA: 9.33 and 15 µL; 2% bis-AA: 1.88 and 14.40 µL; 2 mg/mL streptavidin-AA: 3.33 and 3.33 µL; 10× PBS: 5 and 5 µL; deionized milli-Q water: 30 and 11.17 µL; TEMED: 0.1 and 0.1 µL; 10% APS: 1 and 1 µL. The cured PAA sandwiches were incubated in room temperature deionized water for 1 h for hypotonic coverglass release from PAA gel. After coverglass removal, dish-bound gels retained fluorescent α-collagen-1 Ab μ-patterns. Fourth, the resulting micropatterns or nanopatterns were incubated with 1 mg/mL rat monomeric collagen-1 (Corning, NY, Cat#354249) in cold PBS (4 °C, overnight), rinsed, and used for experiments. Identically, E-cadherin PAA micropatterns were manufactured by transferring biotin+fluorophore-conjugated α-Fc_γ_ Fab (AffiniPure, Jackson Immunoresearch, West Grove, PA, Cat#109-007-008, RRID:AB_2632440) at a concentration of 0.2 mg/mL via microstamping or nanostamping onto PAA patterns. Fab-micropatterned PAA gels then were incubated with human E-cadherin-Fc chimera (R&D Systems, Minneapolis, MN; 0.1 mg/mL, Cat#8505-EC-050) in cold PBS (4 °C, overnight). Combined *coll1⊥Ecad* and *coll1⊥coll1* grids were made by orthogonal criss-cross microprinting of the corresponding proteins, followed by incubation with respective ligand(s) as described above.

### Imaging

High- and super-resolution 3D and 2D imaging for cell morphometric and morpho-mechanical analysis were performed on a Nikon TiE stand with an A1Rsi Confocal scan head, powered by NIS-Elements Confocal software (Nikon, Japan). Objectives used were PlanApo VC ×20/0.75 NA and PlanApo VC 60xWI/1.20NA and excitation was provided sequentially by 405, 488, and 561 nm lasers. Fluorescence was collected through a 1.2-AU pinhole using emission filters of 425–475, 500–550, and 570–620 nm. Pixel size was adjusted to Nyquist sampling (voxel size *x*,*y*,*z* for the ×20 objective; *j*,*k*,*l* for the ×60 objective). Morpho-mechanical and morphometric analysis was performed automatically and/or manually utilizing NIS-Elements Advanced Research software (Nikon, Japan) as an integral part of the data analysis streamline microscopy-to-measurement-to-analysis. Video sequences were also analyzed with ImageJ’s stacks plug-in. Additionally, live cell imaging microscopy experiments were performed on an Olympus X81 (Olympus, Japan) microscopy inside a temperature (37 °C), CO_2_ (5%), and humidity-controlled chamber at ×20 magnification. Brightfield and fluorescent images were obtained every 2 min using the MetaMorph software (Molecular Devices, USA). Composite 2D/3D cells+micropattern images were reconstructed and assembled using NIS-Elements AR and linear image parametric adjustments. Figures were composed from unmodified 3D reconstructions captures utilizing NIS-Elements AR-generated TIFF images with Adobe Illustrator CC 2017 (Adobe).

### Cell protrusion assays

We utilized the triple-negative human breast adenocarcinoma cell line MDA-MB-468 ATCC® HTB-132™ as a model that features both mesenchymal and epithelial phenotypes, is invasive, and expresses E-cadherin. MDA-MB-231 cells (ATCC^®^ HTB-26™) feature an epithelial-to-mesenchymal phenotype, are invasive and metastatic, and do not express E-cadherin and were used to validate collagen and E-cadherin platforms. Both lines were freshly obtained and validated from the ATCC cell bank at the start of these studies and were used within ten passages from initial cultures, with no deviation in phenotype while remaining free of Mycoplasma, for all experiments. For cell culture and during experiments, cells were maintained in Dulbecco’s modified Eagle’s medium (DMEM) with 3.5 g/L d-glucose, L-glutamine, 110 mg/L sodium pyruvate (Corning Cellgro^®^, Cat#10-013-CV), and 10% heat-inactivated fetal bovine serum (HyClone^®^, Cat#SH30910.03). Pharmacological inhibitors were administered 5–10 min prior to cell interactions with contact guidance cues on the micropatterns and were maintained throughout the course of the experiments. Similarly, control groups were pretreated and then incubated with corresponding amounts of vehicle solvent (e.g., dimethyl sulfoxide). All drug concentrations were optimized to the following values: (−)-blebbistatin (Sigma-Aldrich, Cat#203391, 50 μM), nocodazole (AbCam, Cat#ab120630, 50 μM), CK666 (Tocris, Cat#3950, 50 μM), smifH2 (AbCam, Cat#ab218296, 25 nM), and FAK inhibitor 14 (AbCam, Cat#ab144503, 50 μM). Arp2/3 and FAK results were confirmed using a second inhibitor CK869 (AbCam, Cat#ab218109) and PF573228 (Tocris, Cat#PF 573228), respectively, with distinct mechanisms of action, in a subset of experiments. Before utilization, final concentration, drug culture medium solutions were incubated 20 min in 37 °C water bath to ensure that they were fully dissolved and then the solutions were filtered through 0.22-μm Millex®GP (Millipore, Carrigtwohill, Co, Cork, Ireland, Cat#SLGP033RB). Each cell protrusion assay run lasted for ~1 h. Samples then were fixed with cold methanol for MT visualization (−20 °C, 5 min) or with 3% paraformaldehyde (PFA) + 0.5% glutaraldehyde in cytoskeleton buffer for curvature radius measurement as described in previous reports. In all other cases, we utilized cold DMEM with 4% PFA, followed by 0.1% Triton 100-X in 1% bovine serum albumin (BSA) PBS^35^. F-actin was stained with fluorescent phalloidin (Alexa Fluor™ phalloidin conjugates, AbCam, Cat#ab176759 and Cat#ab176753; 10 U/mL in 1% BSA PBS), chromatin was labeled with Hoechst solution (1:1000), paxillin was immunostained with mouse monoclonal antibody (mAb; BD Biosciences, Cat#610052; RRID:AB_397464; 5 μg/mL in 1% BSA PBS, 1 h incubation), and MTs were stained with Alexa Fluor®-conjugated rat mAb (AbCam, Cat#ab197737; 5 μg/mL in 1% BSA PBS, 1 h incubation) or with rat mAb (AbCam, Cat#ab6160; RRID:AB_305328; 5 μg/mL in 1% BSA PBS, 1 h incubation). Cell spreading/protrusion lengths *L* along each ligand direction was measured from end-to-end as shown in Fig. 1. Poorly spread cells were measured only if they developed minimal protrusions along either axis. Cell edge curvature radii were measured by finding corresponding cell-edge-conforming circles with the three-points method as shown in Fig. 1.

### Cell traction forces analysis

For tracking deformation in PAA gels^11^ during TFM analysis, we modified patterned PAA platforms by adding 0.2 µm fluorescent nanobeads (Polysciences, Cat#BLI832-1) into PAA solutions before gel polymerization. Before and after removal of cells, images of the PAA micropatterns were taken with live confocal laser scanning at the interface planes between cells and the adhesion ligand patterns. Live cell imaging was performed in a microclimate-controlled stage top incubator (Tokai Hit, Japan) at 37 °C in 5% CO_2_. Bead displacements and corresponding traction forces fields were calculated using an iterative particle image velocimetry algorithm and an unconstrained Fourier transform traction cytometry algorithm, respectively (ImageJ plugins).

### Statistical analysis

Multiple groups were compared by analysis of variance, followed by Tukey post hoc analysis. Pairwise comparisons were analyzed using *t* test. Statistical analysis was performed using either KaleidaGraph 4.5.3 (Synergy Software) or Prism 7b (GraphPad Software, Inc). Sample size for each comparison is reported in the corresponding plots. Data are shown as mean ± s.d.

### Reporting Summary

Further information on research design is available in the Nature Research Reporting Summary linked to this article.

## Electronic supplementary material

Reporting Summary

Supplementary Information

Peer Review File

Description of Additional Supplementary Files

Supplementary Movie 1

Supplementary Movie 2

Supplemenatry Movie 3

Supplementary Movie 4

Supplementary Movie 5

Supplementary Movie 6

Supplementary Movie 7

Supplementary Movie 8

Supplementary Movie 9

Supplementary Movie 10

Supplementary Movie 11

Supplementary Movie 12

## Data Availability

The authors declare that all data supporting the findings of this study are available within the paper and its Supplementary Information files or from the corresponding author upon reasonable request.
