# Supplementary material for: Bimodal sensing of guidance cues in mechanically distinct microenvironments
Source: Nat Commun. 2018 Nov 20;9:4891. doi: 10.1038/s41467-018-07290-y (PMC6244288; doi:10.1038/s41467-018-07290-y)
Supplement: Supplementary file 1 — Reporting Summary [file 41467_2018_7290_MOESM1_ESM.pdf]

nature research

Corresponding author(s): Paolo P. Provenzano

Reporting Summary

Nature Research wishes to improve the reproducibility of the work that we publish. This form provides structure for consistency and transparency in reporting. For further information on Nature Research policies, see Authors & Referees and the Editorial Policy Checklist.

Statistical parameters

When statistical analyses are reported, confirm that the following items are present in the relevant location (e.g. figure legend, table legend, main text, or Methods section).

n/a	Confirmed	
☐	☒	The exact sample size (*n*) for each experimental group/condition, given as a discrete number and unit of measurement
☐	☒	An indication of whether measurements were taken from distinct samples or whether the same sample was measured repeatedly
☐	☒	The statistical test(s) used AND whether they are one- or two-sided<br>*Only common tests should be described solely by name; describe more complex techniques in the Methods section.*
☒	☐	A description of all covariates tested
☒	☐	A description of any assumptions or corrections, such as tests of normality and adjustment for multiple comparisons
☐	☒	A full description of the statistics including central tendency (e.g. means) or other basic estimates (e.g. regression coefficient) AND variation (e.g. standard deviation) or associated estimates of uncertainty (e.g. confidence intervals)
☒	☐	For null hypothesis testing, the test statistic (e.g. *F*, *t*, *r*) with confidence intervals, effect sizes, degrees of freedom and *P* value noted<br>*Give P values as exact values whenever suitable.*
☒	☐	For Bayesian analysis, information on the choice of priors and Markov chain Monte Carlo settings
☒	☐	For hierarchical and complex designs, identification of the appropriate level for tests and full reporting of outcomes
☒	☐	Estimates of effect sizes (e.g. Cohen's *d*, Pearson's *r*), indicating how they were calculated
☐	☒	Clearly defined error bars<br>*State explicitly what error bars represent (e.g. SD, SE, CI)*

Our web collection on statistics for biologists may be useful.

Software and code

Policy information about availability of computer code

Data collection	All software used in the study is commercially or publically available<br>1. Microscopy imaging was powered by NIS-Elements Confocal software, v.4.5 (Nikon, Japan).<br>2. Live cell imaging was powered by MetaMorph v.7.8
Data analysis	1. Image analysis and measurements with NIS-Elements Advanced Research software, v.4.5 (Nikon, Japan)<br>2. Traction force analysis with iterative particle image velocimetry (PIV by Tseng, Q) algorithm plug-in for ImageJ.<br>3. Stack analysis and traction force analysis performed on FiJi platform (ImageJ) v.2.0.0<br>4. Data tabulation and elements of data analysis with MS Excel v.16.16.1<br>5. For statistical analysis we used KaleidaGraph v.4.5.3 (Synergy Software) and Prism v.7b (GraphPad Software, Inc)

For manuscripts utilizing custom algorithms or software that are central to the research but not yet described in published literature, software must be made available to editors/reviewers upon request. We strongly encourage code deposition in a community repository (e.g. GitHub). See the Nature Research guidelines for submitting code & software for further information.

Data

Policy information about availability of data

All manuscripts must include a data availability statement. This statement should provide the following information, where applicable:

- Accession codes, unique identifiers, or web links for publicly available datasets
- A list of figures that have associated raw data
- A description of any restrictions on data availability

The authors declare that all data supporting the findings of this study are available within the paper and its Supplementary Information Files or from the authors upon reasonable requests.

Field-specific reporting

Please select the best fit for your research. If you are not sure, read the appropriate sections before making your selection.

☒ Life sciences ☐ Behavioural & social sciences ☐ Ecological, evolutionary & environmental sciences

For a reference copy of the document with all sections, see nature.com/authors/policies/ReportingSummary-flat.pdf

Life sciences study design

All studies must disclose on these points even when the disclosure is negative.

Sample size	Sample sizes varied from ~50 through ~1700, depending on the availability of larger sample sets and inherent to each method technical limitations. Technically complex measurements were optimized to the sample sizes of ~20 for each replicate, however, only if measurements results grouped well, otherwise the sample sizes were increased to ~100 as this number captured trends well.
Data exclusions	No data was excluded.
Replication	Several replicates (independent experiments) were conducted for each data set. Number of replicates varied from N=3 to N=5. Each replicate included at least 2, but usually 3 parallel samples for each condition.
Randomization	Randomization was ensured by the blind picking of the regions of the interest (ROI), i.e. the microscopy of the samples was performed following the single preprogrammed algorithm of ROI choice across all samples and experiments after defining the true geometric centre of the sample.
Blinding	Blinding was ensured by randomization of choice of the region of the interest (ROI), within each ROI the entire cell population was measured, as long as cells were attached and developed any minimal adhesion and spreading-relevant phenotype.

Reporting for specific materials, systems and methods

Materials & experimental systems

n/a	Involved in the study
☒	☐ Unique biological materials
☐	☒ Antibodies
☐	☒ Eukaryotic cell lines
☒	☐ Palaeontology
☒	☐ Animals and other organisms
☒	☐ Human research participants

Methods

n/a	Involved in the study
☒	☐ ChIP-seq
☒	☐ Flow cytometry
☒	☐ MRI-based neuroimaging

Antibodies

Antibodies used	1. α-Collagen-1 rabbit pAb (AbCam, Cambridge, UK, Cat#ab34710; RRID:AB_731684), Lot:GR3217975-2<br>2. α-Fcγ Fab (AffiniPure, Jackson Immunoresearch, West Grove, PA, Cat#109-007-008, RRID:AB_2632440), Lot:126686<br>3. α-Paxillin mouse mAb, clone "349/Paxillin" (BD Biosciences, Cat#610052; RRID:AB_397464), Lots:5204619 and 7201859<br>4. α-Tubulin rat mAb (AbCam, Cat#ab6160; RRID:AB_305328), Lot:GR3208838-3<br>5. α-Tubulin Alexa Fluor®-conjugated rat mAb (AbCam, Cat#ab197737), Lot:GR222196-1
Validation	1. α-Collagen-1 rabbit pAb commerically tested for IHC-Fr, indirect ELISA, Western Blot (See: Tillgren V et al. J Biol Chem

290:918-25; 2015), IHC-P, ELISA. ICC/IF and IP. See https://www.abcam.com/collagen-i-antibody-ab34710.html

2. α-Fcγ Fab (AffiniPure) was tested for human E-cadherin-Fc chimera protein adsorption activity, with the following immunostaining of the E-cadherin extracellular domains. See Supplementary Figure 9c.

3. α-Paxillin mouse mAb (BD Biosciences) is commercially routinely tested by Western Blot analysis, see http://www.bdbiosciences.com/us/reagents/research/antibodies-buffers/cell-biology-reagents/cell-biology-antibodies/purified-mouse-anti-paxillin-349paxillin/p/610052

4. α-Tubulin rat mAb (AbCam) commercially tested for IHC-Fr, IP, RIA, Western Blot, Flow Cytometry, ICC/IF, IHC (PFA), IHC-P, IHC-Wholempunt, IHC (Methanol, see PubMed:16943269). See https://www.abcam.com/tubulin-antibody-yl12-loading-control-ab6160.html

5. α-Tubulin Alexa Fluor®-conjugated rat mAb (AbCam) was commercially tested for ICC/IF.
See https://www.abcam.com/tubulin-antibody-yl12-alexa-fluor-488-ab197737.html

Eukaryotic cell lines

Policy information about cell lines

Cell line source(s)	Cell line MDA-MB-468 ATCC® HTB-132™ was obtained freshly directly from ATCC® cell bank.
Authentication	Cell lines were freshly obtained directly from ATCC® cell bank, where they were validated, at the start of these studies and were used within 10 passages from initial cultures, with no deviation in phenotype.
Mycoplasma contamination	Cell line was continuously monitored for Mycoplasma.
Commonly misidentified lines (See ICLAC register)	None of the commonly misidentified cell lines was used in this study.
