# Supplementary material for: Bimodal sensing of guidance cues in mechanically distinct microenvironments
Source: Nat Commun. 2018 Nov 20;9:4891. doi: 10.1038/s41467-018-07290-y (PMC6244288; doi:10.1038/s41467-018-07290-y)
Supplement: Supplementary file 2 — Supplementary Information [file 41467_2018_7290_MOESM2_ESM.pdf]

Supplementary Information:

Bimodal Sensing of Guidance Cues in Mechanically Distinct Microenvironments

Tabdanov et al.

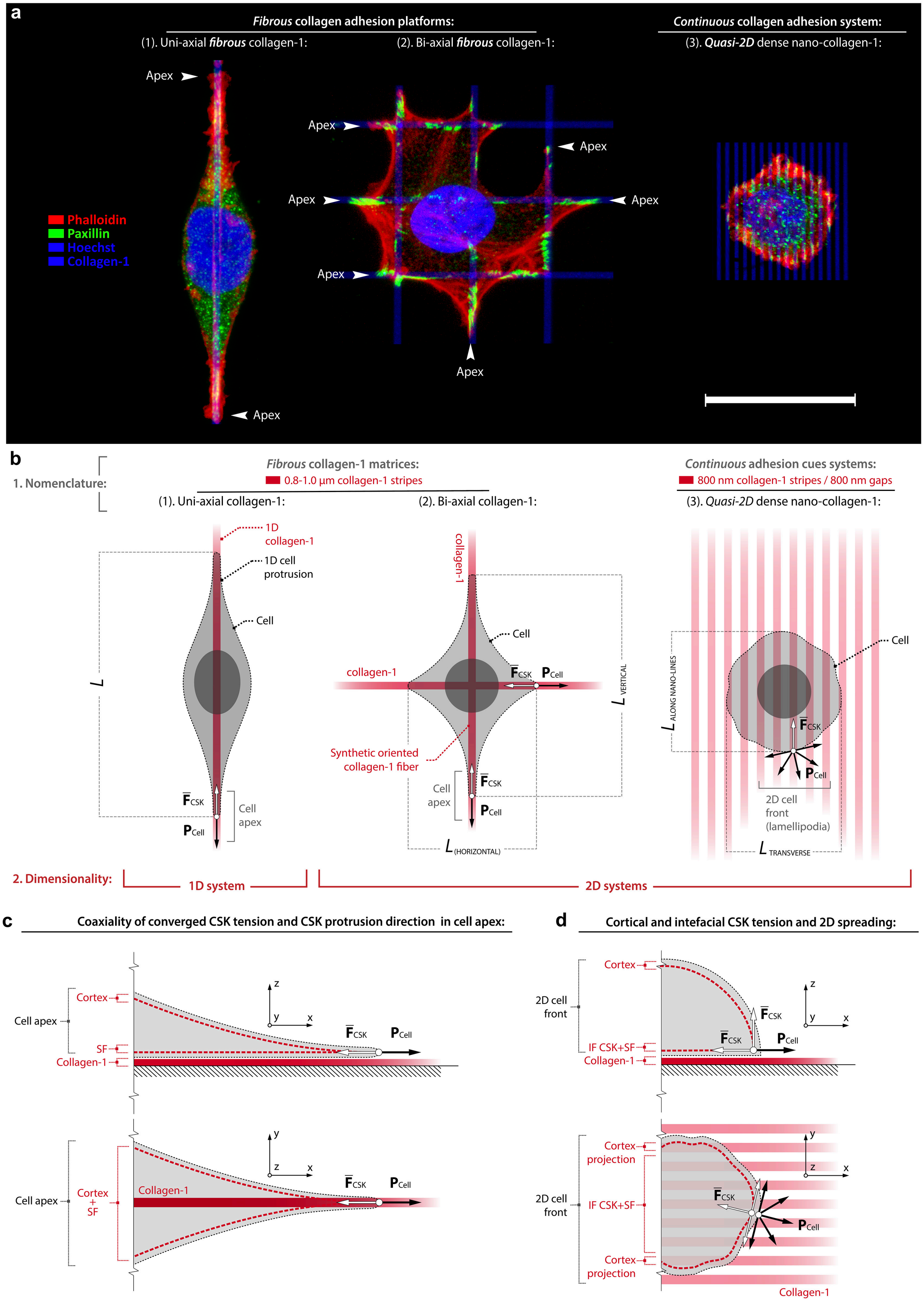
a
Fibrous collagen adhesion platforms:
(1). Uni-axial fibrous collagen-1:
(2). Bi-axial fibrous collagen-1:
Continuous collagen adhesion system:
(3). Quasi-2D dense nano-collagen-1:
Apex
Phalloidin
Paxillin
Hoechst
Collagen-1
b
1. Nomenclature:
Fibrous collagen-1 matrices:
0.8-1.0 µm collagen-1 stripes
(1). Uni-axial collagen-1:
(2). Bi-axial collagen-1:
Continuous adhesion cues systems:
800 nm collagen-1 stripes / 800 nm gaps
(3). Quasi-2D dense nano-collagen-1:
1D collagen-1
1D cell protrusion
Cell
L
F̄CSK
PCell
Cell apex
collagen-1
L VERTICAL
L (HORIZONTAL)
Synthetic oriented collagen-1 fiber
L ALONG NANO-LINES
L TRANSVERSE
2D cell front (lamellipodia)
2. Dimensionality:
1D system
2D systems
c
Coaxiality of converged CSK tension and CSK protrusion direction in cell apex:
Cell apex
Cortex
SF
Collagen-1
Cortex + SF
z
y
x
d
Cortical and intefacial CSK tension and 2D spreading:
2D cell front
IF CSK+SF
Cortex projection

Supplementary Figure 1 | Comparison of *fibrous* uniaxial (1D) and biaxial (2D) guidance cues to flat (~2D) collagen nanolines

(**a**) MDA-MB-468 cells on *fibrous* patterns of **(1)** single 1D, i.e. uniaxial, collagen guidance cues, **(2)** orthogonally crossed biaxial, i.e. 2D synthetic *fibrous*, 0.8-1 μm collagen networks, and **(3)** collagen nanolines (800 nm-wide collagen lines and alternating 800 nm gaps) representing quasi-2D substrates on flat elastic PAA gel surfaces, scale bar - 30 μm. Nano-patterns provide sufficient adhesion density for multidirectional lamellipodia to form. Cells on *fibrous* uni- and biaxial cues develop longer, phenotypically mesenchymal cell-like, protrusions on either uniaxial and biaxial cues, respectively, compared to quasi-2D collagen nanolines, where cells remain largely spread in all directions, a more epithelial phenotype. *Note that all cells and corresponding collagen patterns are shown at the same magnification.*

(**b**) Schematics, metrics, and related nomenclature for cell sensing of guidance cues: **1 and 2 -** Nomenclature and dimensionality of the cell adhesion systems: *Fibrous* cues are divided into **(1)** uniaxial lines and **(2)** biaxial grids. While the uniaxial system corresponds to an aligned fiber, the biaxial system constitutes a 2D *fibrous* system that facilitates bi-directional cell spreading and represents more isotopic collagen matrices. **(3)** The more continuous, quasi-2D system corresponds to a separate group of adhesion systems that facilitates multi-axial 2D cell protrusions. Cell protrusive spreading on uniaxial collagen **(1)** is measured as the cell length between opposed cell apices, while cell protrusive spreading on biaxial collagen **(2)** is measured as vertical and horizontal cell lengths. **(3)** Globally, MDA-MB-468 spreading on collagen nanolines displays no alignment to the nanoline directionality. Cell spreading measurements were performed along the nano-lines (*L* along nano-lines) and in the transverse direction to the nanolines ($L_{\text{TRANSVERSE}}$). *Fibrous* uni- and biaxial cues induce narrow or non-lamellar lamellipodia cell protrusion apices. Guided by collagen lines, apical protrusions converge global cell cytoskeletal (CSK) tensile forces ($\mathbf{F}_{\text{CSK}}$) into ~uniaxial tension by virtue of the apex geometry, which is coaxial to the direction of cell protrusion ($\mathbf{P}_{\text{Cell}}$) in contrast with multidirectional cell spreading on nanolines (*right*).

(**c**) Convergence of CSK tension on cell apices is global and includes both stress-fibers (SF) and the CSK cortex. On *fibrous* (e.g. uniaxial) guidance cues, the geometry of the cell protrusion unites the cortical and tensile adhesion cytoskeleton into a single entity.

(**d**) Multi-directional cell protrusion on quasi-2D collagen nanolines induces a more circular MDA-MB-468 morphology despite ECM directionality (as observed from analysis of 3D cell volumes for cells shown in **a**). On nanolines, lack of coaxiality between the interfacial cytoskeletal (IF CSK) tension and cell spreading $\mathbf{P}_{\text{Cell}}$ can be additionally complicated by more off-plane cortical tension F_{CSK}, which are conditional, as they depend on the lack of cortex-IF CSK convergence, which in turn depends on the degree of cell spreading/flattening. Thus, biaxial guidance cues systems to mimic multidirectional cues from physiologically relevant fibrous network provide for direct comparison to the uniaxial adhesion systems for contact guidance, which cannot be adequately compared to continuous or quasi-2D adhesion systems for the questions asked in the current study, as they are governed by principally different CSK architectures.

Scale bar - 30 μm

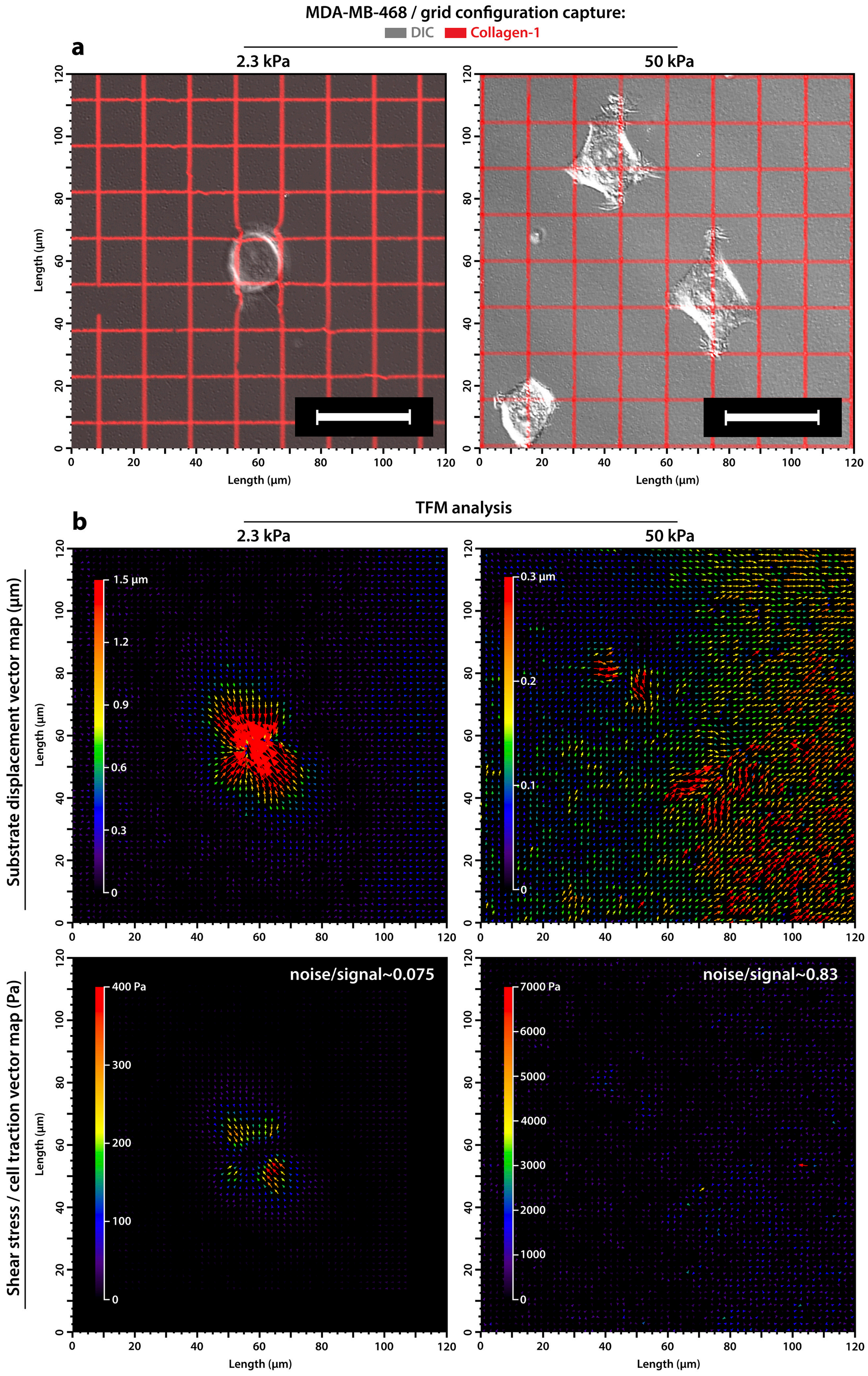

MDA-MB-468 / grid configuration capture:
DIC
Collagen-1
a
2.3 kPa
50 kPa
Length (μm)
b
TFM analysis
Substrate displacement vector map (μm)
1.5 μm
0.3 μm
Shear stress / cell traction vector map (Pa)
400 Pa
7000 Pa
noise/signal~0.075
noise/signal~0.83

Supplementary Figure 2 | Stiff (50 kPa) substrates are outside of the optimal stiffness range suitable for traction force analysis with MDA-MB-468 cells

(**a**) DIC (cells) and fluorescence (collagen; *red*) of MDA-MB-468 carcinoma cells on soft (2.3 kPa) and stiff (50 kPa) substrates, demonstrating well-detectable deformation of soft, but not stiff substrates.

(**b**) Substrate elastic deformation (μm) and cell traction stress (Pa) vector maps. The color ranges encode the scalar map for local displacement (μm) and shear stress (Pa). Traction force map for 2.3 kPa collagen grids show a low noise-to-signal ratio (~0.075) while cells on stiff, 50 kPa collagen grids show a high noise-to-signal ratio (~0.83) due to low deformations that are not sufficient to accurately solve the inverse traction force problem. Scale bars - 30 μm.

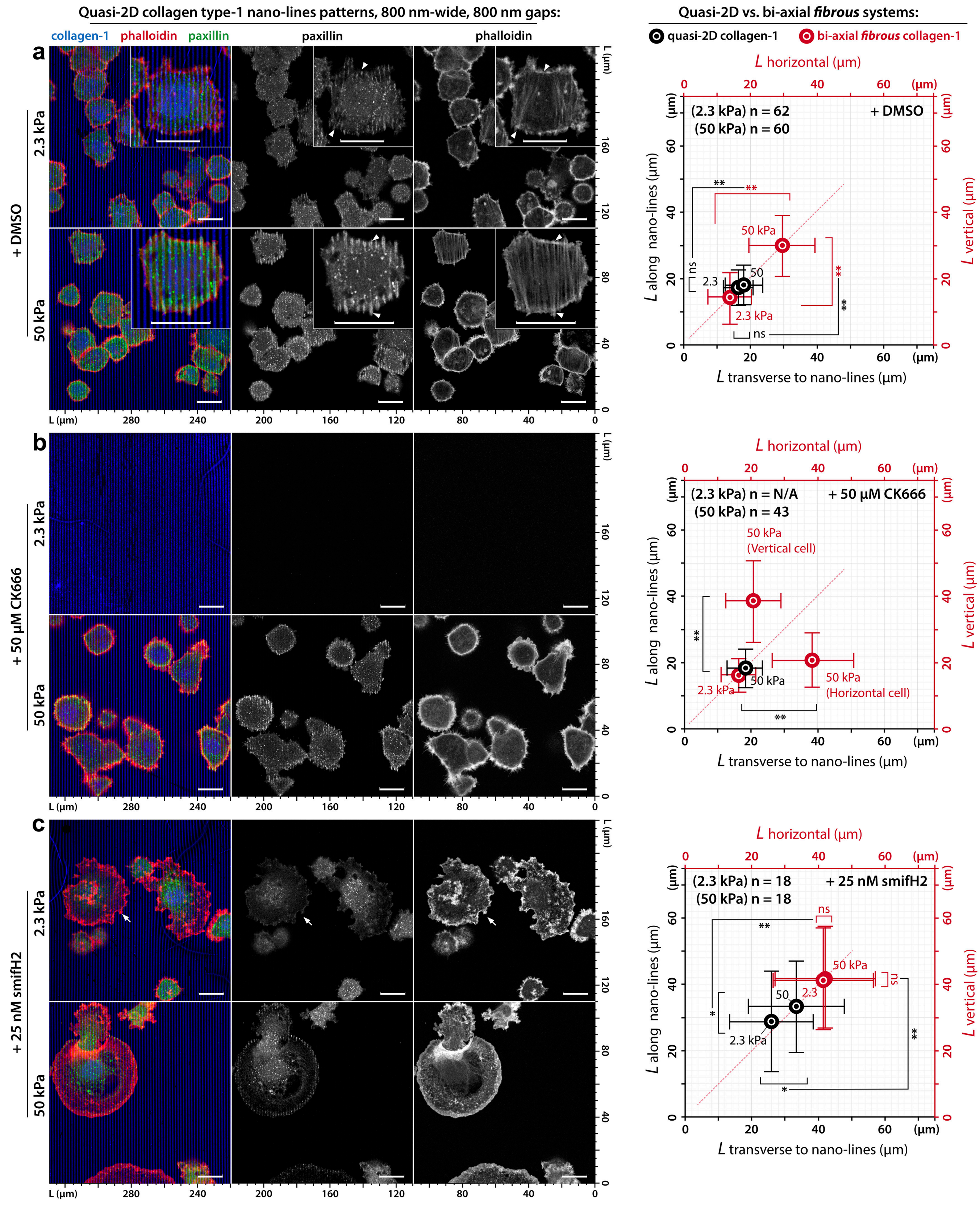
Quasi-2D collagen type-1 nano-lines patterns, 800 nm-wide, 800 nm gaps:
collagen-1 phalloidin paxillin
paxillin
phalloidin
Quasi-2D vs. bi-axial fibrous systems:
quasi-2D collagen-1
bi-axial fibrous collagen-1
a
b
c
+ DMSO
2.3 kPa
50 kPa
+ 50 µM CK666
+ 25 nM smifH2
L (µm)
280
240
200
160
120
80
40
0
L horizontal (µm)
L vertical (µm)
L along nano-lines (µm)
L transverse to nano-lines (µm)
(2.3 kPa) n = 62
(50 kPa) n = 60
+ DMSO
(2.3 kPa) n = N/A
(50 kPa) n = 43
+ 50 µM CK666
50 kPa (Vertical cell)
50 kPa (Horizontal cell)
(2.3 kPa) n = 18
(50 kPa) n = 18
+ 25 nM smifH2
2.3 kPa
50 kPa
ns
**
*

Supplementary Figure 3 | Response to quasi-2D collagen nanoline guidance cues

(**a-c**) Visual (*left panels*) and numerical (*right panels*) comparison of MDA-MB-468 behavior on soft (2.3 kPa) vs. stiff (50 kPa) collagen substrates in control (+DMSO), Arp2/3 suppression (+50 µM CK666) and formin suppression (+25 nM smifH2) conditions in arrayed matrix-form. Cell protrusion analysis was performed as described in **Supplementary Figure 1**.

(**a**) MDA-MB-468 cells in the presence of vehicle (DMSO) on collagen nanolines show no detectable morphological response to the underlying collagen guidance cues or mechanics unlike *fibrous* biaxial cues that produce strong mechano-responsiveness, developing greater vertical and horizontal apical protrusion lengths on the stiffer guidance cues (*red*). Furthermore, compared to cells on *fibrous* biaxial grids and uniaxial lines (**Figures 2** and **3**), nanolines induce significantly less cell protrusion in both directions. . These considerable differences in the cellular response to the two distinct types of 2D guidance cue systems (i.e. quasi-2D vs. biaxial cues) suggests principally different mechanisms governing cell adhesion and protrusion. Corresponding n values for quasi-2D nanolines platforms are shown on the plot, n values for bi-axial cell lengths data are shown on **Figure 2d**.

(**b**) Suppression of Arp2/3 complex with 50 µM CK666 does not change the degree of cell protrusion compared to control conditions (**a**) for stiff substrates, but completely abrogates cell adhesion to the soft (2.3 kPa) collagen surfaces (**a**). Interestingly, cells on quasi-2D collagen nanolines (*black*) did not develop the uniaxial response to biaxial guidance cues (*red*) observed on stiff substrates (see **Figure 5**). Corresponding n values for quasi-2D nanolines platforms are shown on the plot, n values for bi-axial cell lengths data are shown on **Figure 4a**.

(**c**) Inhibition of formins with 25 nM smifH2 in MDA-MB-468 cells on quasi-2D collagen nanolines develop a lamellar morphology and a greater cell spreading in a mechanosensitive manner at the early stages of cell adhesion, but collapse into a dendritic disarrayed cell architecture within 1 hour post-cell seeding. Corresponding n values for quasi-2D nanolines are shown on the plot, n values for bi-axial cell lengths data are n=21 (2.3 kPa) and n=14 (50 kPa). Scale bars - 15 µm. Number of replicates (independent experiments) for all measurements N=4. Data in the plot panels (*right*) are mean±s.d.; ns indicates no significant difference between groups; multiple groups were compared by ANOVA, followed by the Tukey's post-hoc analysis. Pairwise comparisons were analyzed using a t-test; *p<0.05, **p<0.001.

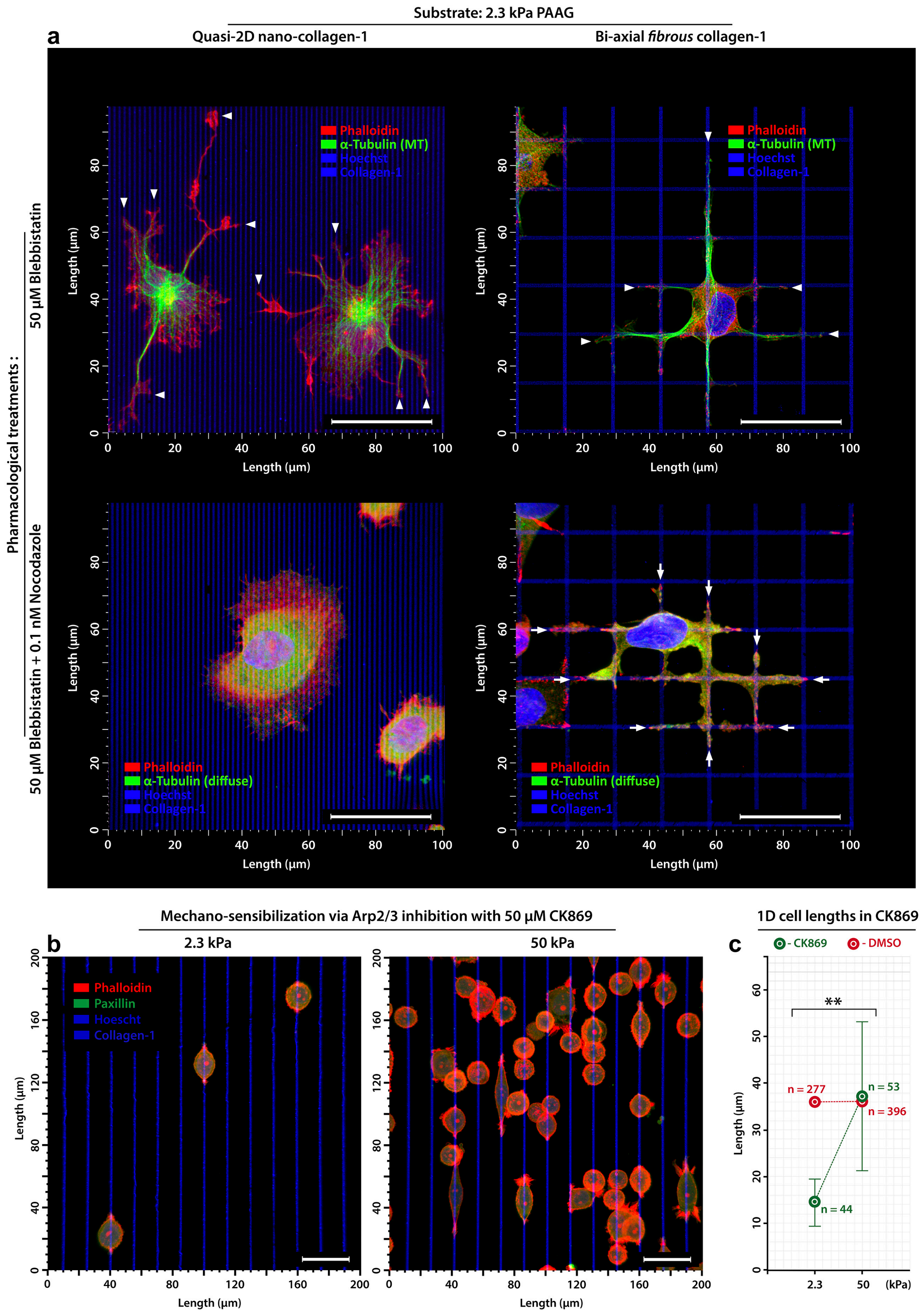
Substrate: 2.3 kPa PAAG
a
Quasi-2D nano-collagen-1
Bi-axial fibrous collagen-1
Pharmacological treatments :
50 μM Blebbistatin
50 μM Blebbistatin + 0.1 nM Nocodazole
Phalloidin
α-Tubulin (MT)
Hoechst
Collagen-1
α-Tubulin (diffuse)
Length (μm)
b
Mechano-sensibilization via Arp2/3 inhibition with 50 μM CK869
2.3 kPa
50 kPa
Phalloidin
Paxillin
Hoescht
Collagen-1
c
1D cell lengths in CK869
- CK869
- DMSO
**
n = 277
n = 53
n = 396
n = 44
(kPa)

Supplementary Figure 4 | (**a**) Intact microtubules (MTs) are crucial for blebbistatin-induced random dendritic cell protrusions on quasi-2D collagen nanolines but are dispensable for guided dendritic protrusion in response to fibrous biaxial cues.MDA-MB-468 cells on soft 2.3 kPa collagen nanolines show random multidirectional dendritic protrusions in the presence of blebbistatin (*white arrowheads*). Destabilization of MTs with nocodazole suppresses dendritic formation and induced an isotropic cell spreading morphology. In response to architecture from biaxial guidance cues, cells develop dendritic protrusions in the presence of blebbistatin irrespective of intact MT (i.e. +/- nocodazole) and with similar cell motility dynamics (see **Movie 7**, cell biaxial dendritic migration in blebbistatin+nocodazole).
(**b**) Validation of the impact of Arp2/3 complex inhibition in MDA-MB-468 cells.Select results obtained by inhibiting Arp2/3 complex with CK666, which inhibits subunits 3 and 4 of Arp2/3, were confirmed with an alternative Arp2/3 inhibitor, CK869, which inhibits subdomain-1 and prevents its association into the Arp2/3 complex. Despite CK869 having different pharmacological kinetics and lower efficiency, similar to CK666 treatment effects, ~50 μM CK869 selectively suppressed uniaxial cell protrusion on soft (2.3 kPa) but not stiff (50 kPa) collagen guidance cues.
Scale bars - 30 μm. Corresponding n values are shown on the plot. Number of replicates (independent experiments) for all measurements N=2. Data in (**c**) are mean±s.d.; ns indicates no significant difference between groups; *p<0.05, **p<0.001.

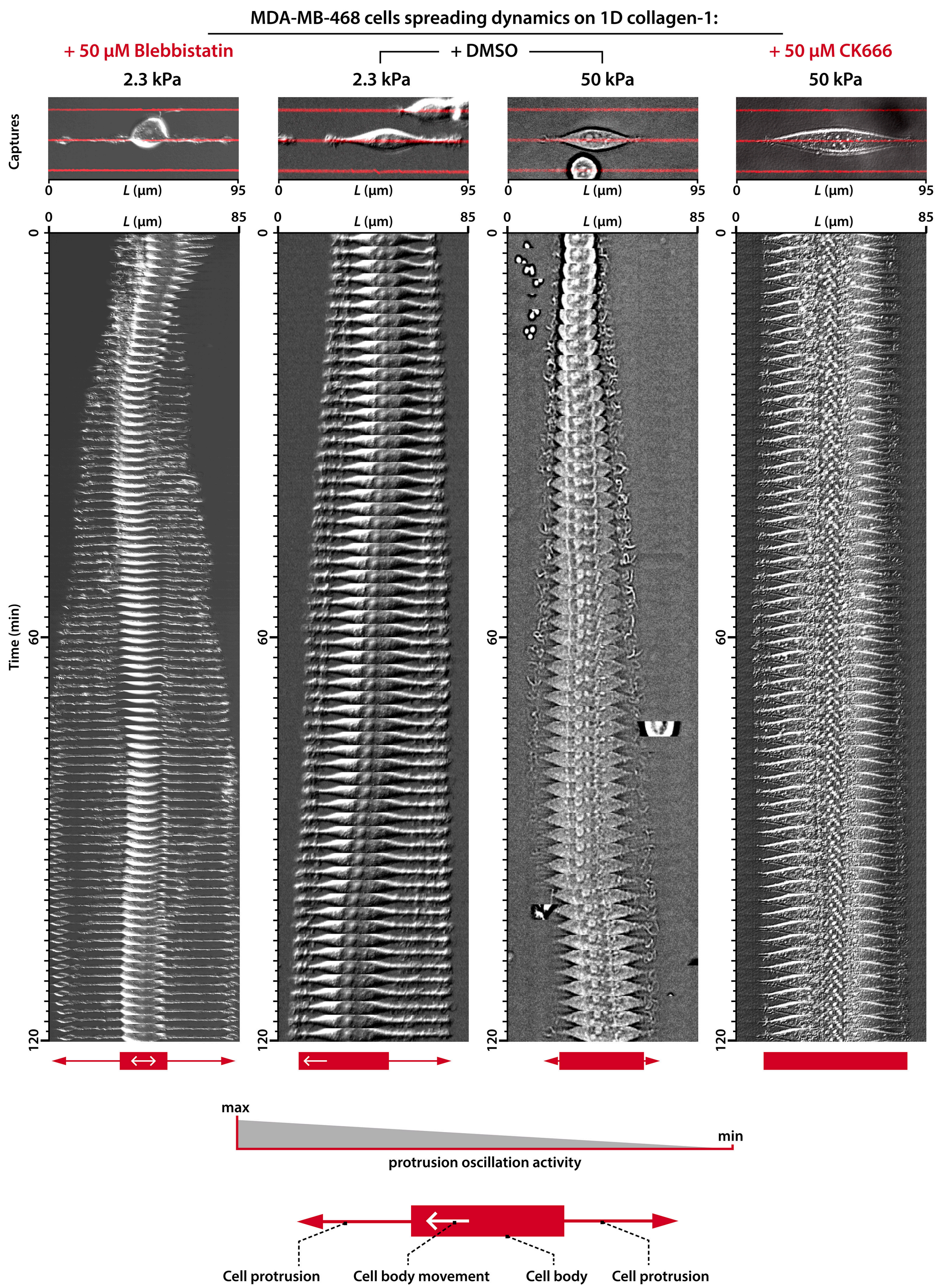
MDA-MB-468 cells spreading dynamics on 1D collagen-1:
+ 50 µM Blebbistatin
+ DMSO
+ 50 µM CK666
2.3 kPa
2.3 kPa
50 kPa
50 kPa
Captures
L (µm)
0
95
85
Time (min)
0
60
120
max
min
protrusion oscillation activity
Cell protrusion
Cell body movement
Cell body
Cell protrusion

Supplementary Figure 5 | MDA-MB-468 cells dynamics on uniaxial collagen guidance cues

Representative analysis of cell spreading on uniaxial collagen guidance cues with blebbistatin treatment on a compliant substrates (2.3 kPa) or CK666 treatment on a stiff substrates (50 kPa) compared to DMSO control conditions. Protrusion oscillatory activity decreases with increasing stiffness and perturbation of actin assembly. With loss of intrinsic cell contractility, or on soft substrates, cell protrusion is smooth over time consistent with our findings of a more fluidic phenotype, in contrast to cells on stiff substrates with more compacted protrusion-retraction behavior. Furthermore, cell oscillatory activity, however, is higher on biaxial guidance cues (**Supplementary Figure 7**), where it is clearly linked to Arp2/3 activity.

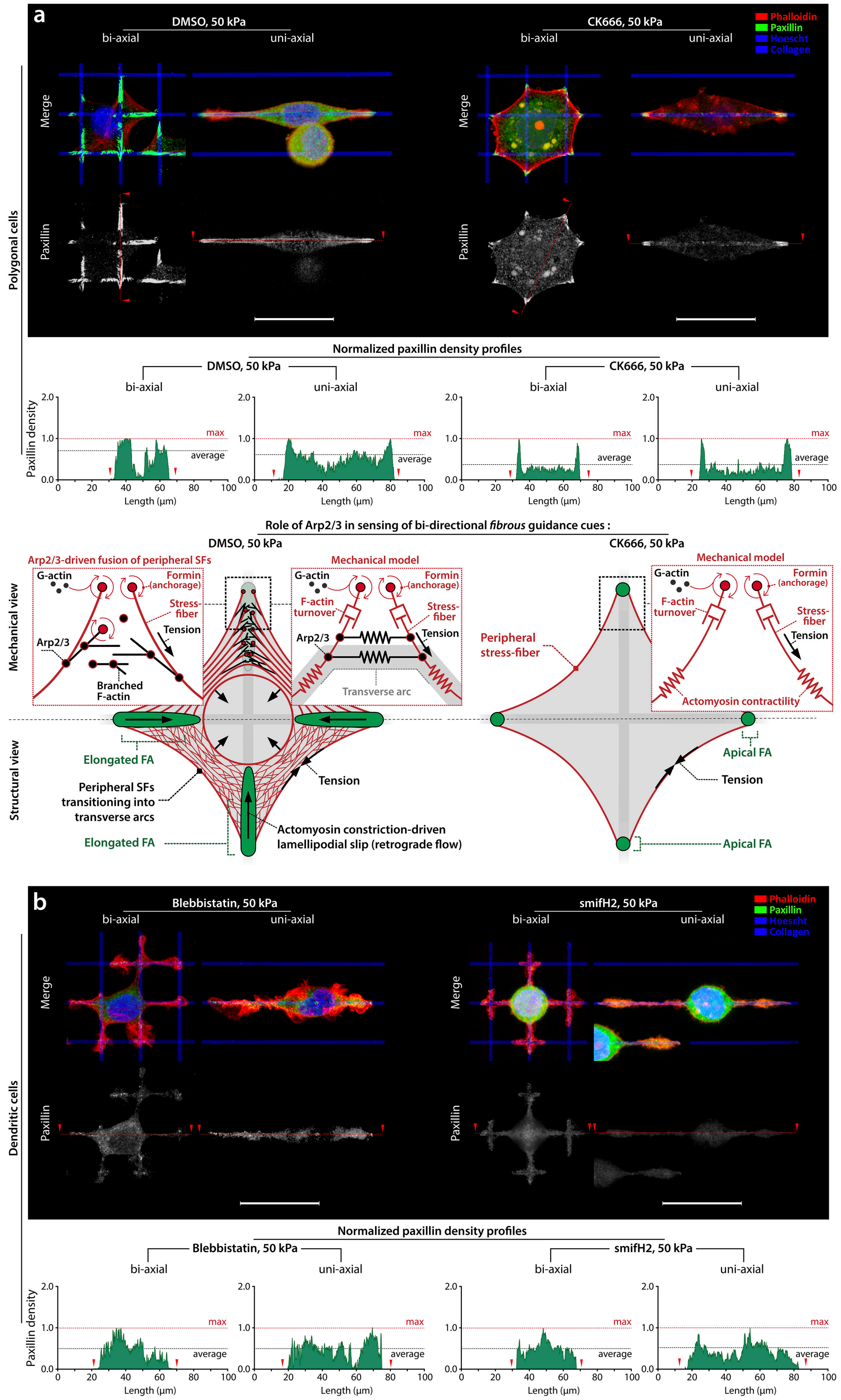

a
DMSO, 50 kPa
bi-axial
uni-axial
CK666, 50 kPa
Phalloidin
Paxillin
Hoescht
Collagen
Polygonal cells
Merge
Paxillin
Normalized paxillin density profiles
Paxillin density
max
average
Length (µm)
Role of Arp2/3 in sensing of bi-directional fibrous guidance cues :
Arp2/3-driven fusion of peripheral SFs
G-actin
Formin (anchorage)
Stress-fiber
Tension
Arp2/3
Branched F-actin
Mechanical model
F-actin turnover
Transverse arc
Peripheral stress-fiber
Actomyosin contractility
Mechanical view
Structural view
Elongated FA
Peripheral SFs transitioning into transverse arcs
Actomyosin constriction-driven lamellipodial slip (retrograde flow)
Apical FA
b
Blebbistatin, 50 kPa
smifH2, 50 kPa
Dendritic cells

Supplementary Figure 6 | Structure of adhesion machinery in MDA-MB-468 cells on stiff guidance cues under control conditions (DMSO) and either myosin, Arp2/3 complex, or formin inhibition

(**a - *top***) Inhibition of Arp2/3 complex alters FA distribution and actin architecture with redistribution FAs strictly to the apical regions (apical FAs) in both uni- and biaxial systems with CK666 treatment (see also **Figure 4f**), in contrast to more prolonged adhesions (elongated FAs) and stress-fibers in control cells (+DMSO, see also **Figures 1** and **3**, **Supplementary Figure 1**). Note, that anisotropy of the adhesion distribution is dictated by the distribution of the cell stresses[1].

(**a - *bottom***) Schematic representation of stress-fibers and FA dynamics in cells under control (+DMSO) and Arp2/3 inhibition (+CK666) conditions. Both cases feature active formins dynamics that facilitate physical anchorage of stress-fibers to FAs and their elongation and turnover[2–5]. Active Arp2/3 facilitates F-actin branching that in turn mediates 1D-lamellipodial dynamics on the collagen lines. Conventionally known F-actin branching and 1D-lamellipodial slip (retrograde flow) facilitate fusion and transitioning of peripheral SFs into the transverse arcs rings[6]. Thus, in our *fibrous* biaxial guidance cue system, actomyosin contractility-driven constriction of transverse arcs drives slippage of transverse arcs inward, establishing the elongated FAs along the span of inner actomyosin network (transverse arcs) that is less aligned toward the boundaries of cell-collagen lines adhesion configuration[6].

(**b**) Low contractility (blebbistatin) or non-contractile (smifH2) cells develop a dendritic morphology phenotype where distribution of FAs is diffuse and irregular in both uni- and biaxial guidance systems.

Scale bars - 30 μm.

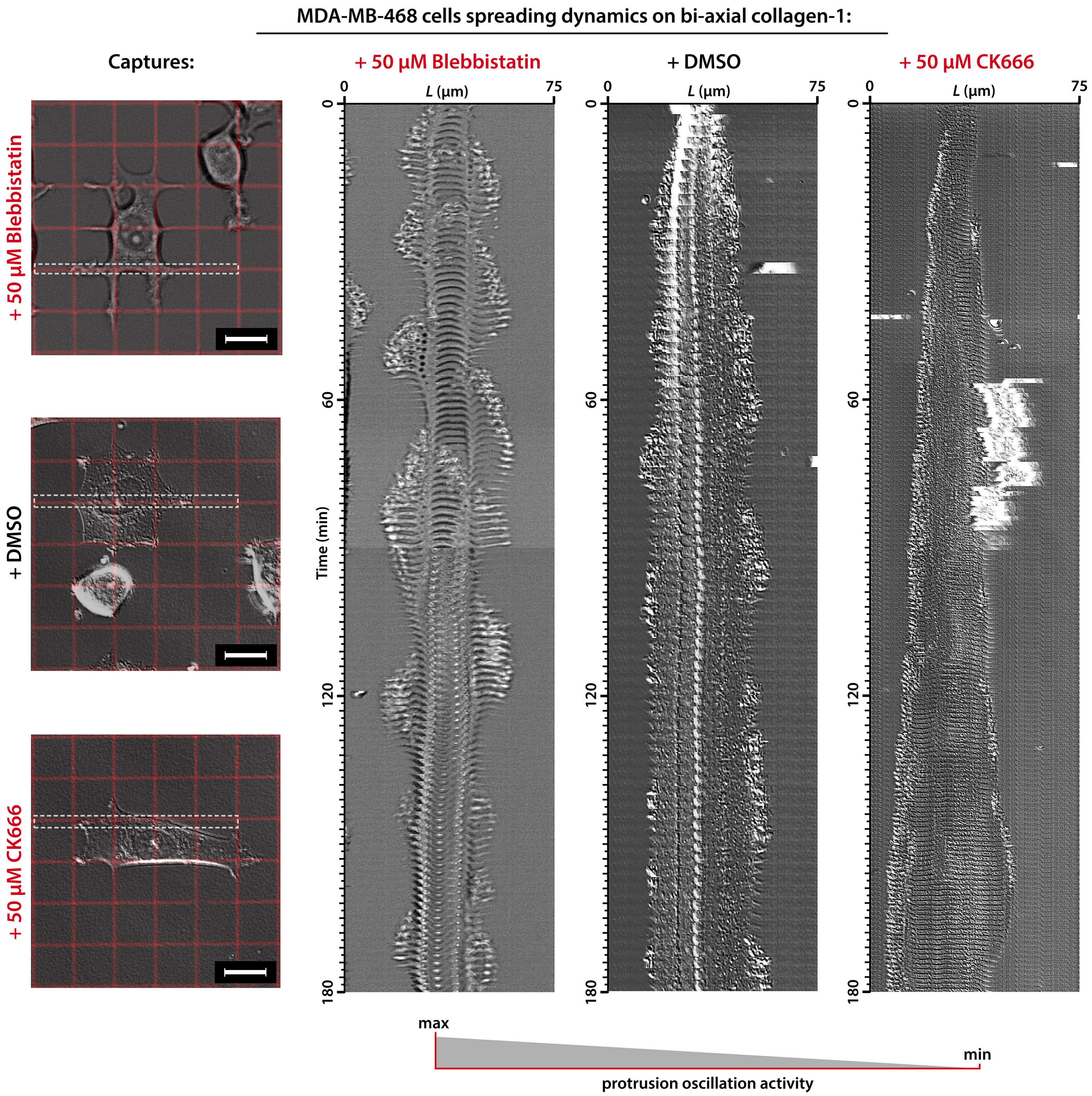

Supplementary Figure 7 | MDA-MB-468 cells dynamics on stiff biaxial collagen guidance cues
Oscillation dynamics of cell apical protrusion-retraction cycles across biaxial guidance cues in the control conditions (+DMSO), low-contractility cell state (+blebbistatin) and during suppression of Arp2/3 complex (+CK666). Note that the oscillation dynamics rate decreases in the following order: low-contractility (high rate) > control (moderate rate) > Arp2/3 inhibition (low rate). Thus, Arp2/3 activity facilitates 1D lamellipodial dynamics (oscillations) that are necessary for the cell probing mode of spreading, whereas inhibition of Arp2/3 activity (+CK666) unmasks the steady dynamics of cell elongation/protrusion, driven by formins (see also Figure 4). Importantly, *tidal* apical oscillations reflect an unstable, transient, cell adhesion mode that occurs in parallel to the steady, formins-dependent, cell protrusion via a stable FA-based mode of cell adhesion and protrusion. Scale bars - 30 μm.

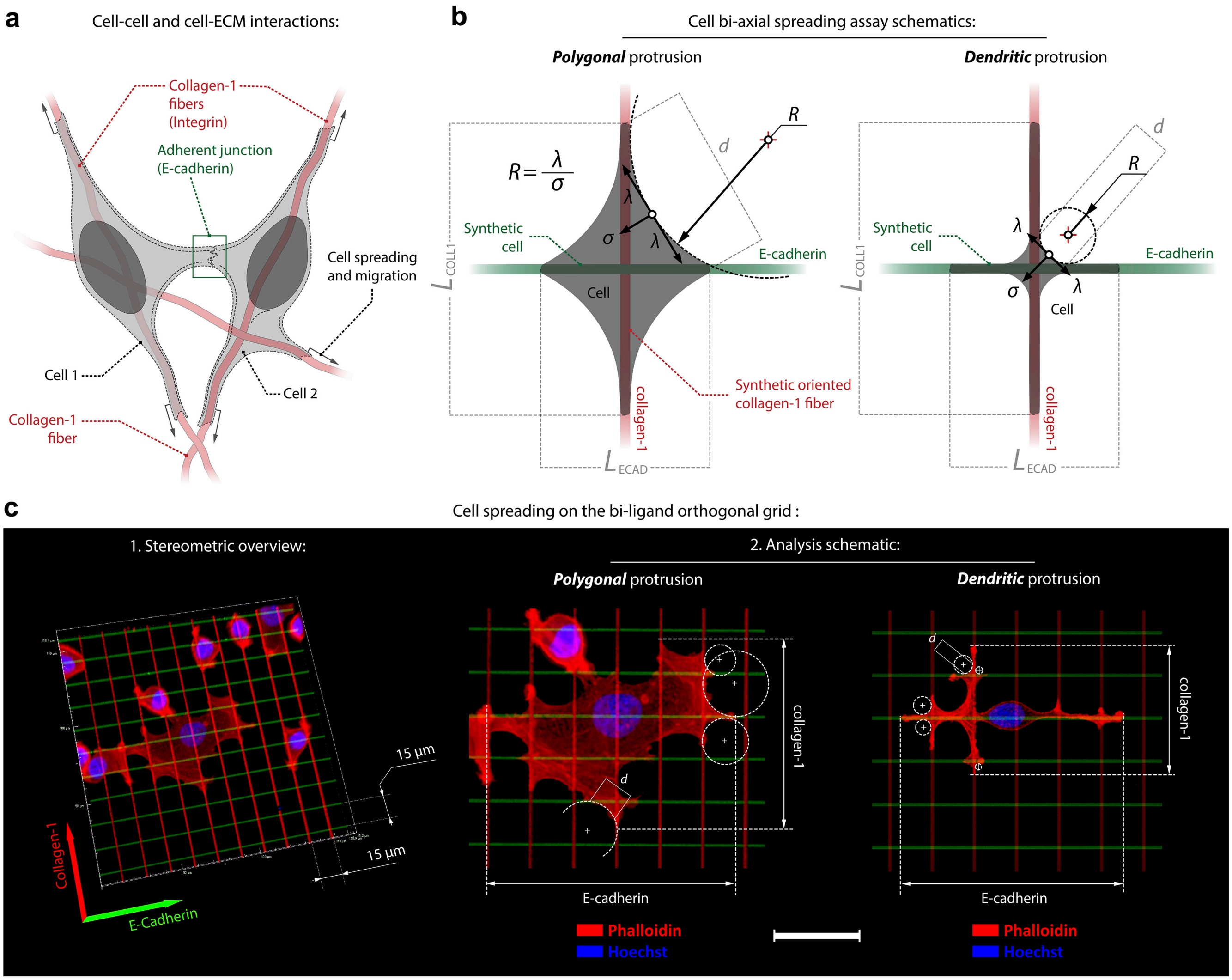

Supplementary Figure 8 | Biomimetic cell contact guidance platform of variable rigidity to study competition between cell-ECM and cell-cell adhesions

(**a**) Schematic of prominent cell adhesion interactions in the 3D cell microenvironment.

(**b**) Schematic of spatially separated and orthogonally oriented lines of collagen (*red*) and E-cadherin (*green*). The orthogonal grid architecture minimizes spatial co-localization of the adhesion ligands and provides minimal co-orientation of cell protrusion along each of the adhesion systems, while retaining their biomechanical coupling[7]. Likewise, lines provide uniaxial cues that mimic interactions with 3D ECM fibers and the cell surface. Free cell edge curvature radii reflect the balance between the internal 2D tension σ and 1D linear cell edge tension λ as related by a 2D Laplace law model following $R=\lambda/\sigma$.

(**c**) Example of cell architecture in response to guidance cues and substrate mechanical properties (1) and phenotypic and mechanical analysis of cells (2). Note, cells in section (2) are shown at the same magnification. Scale bar - 30 µm.

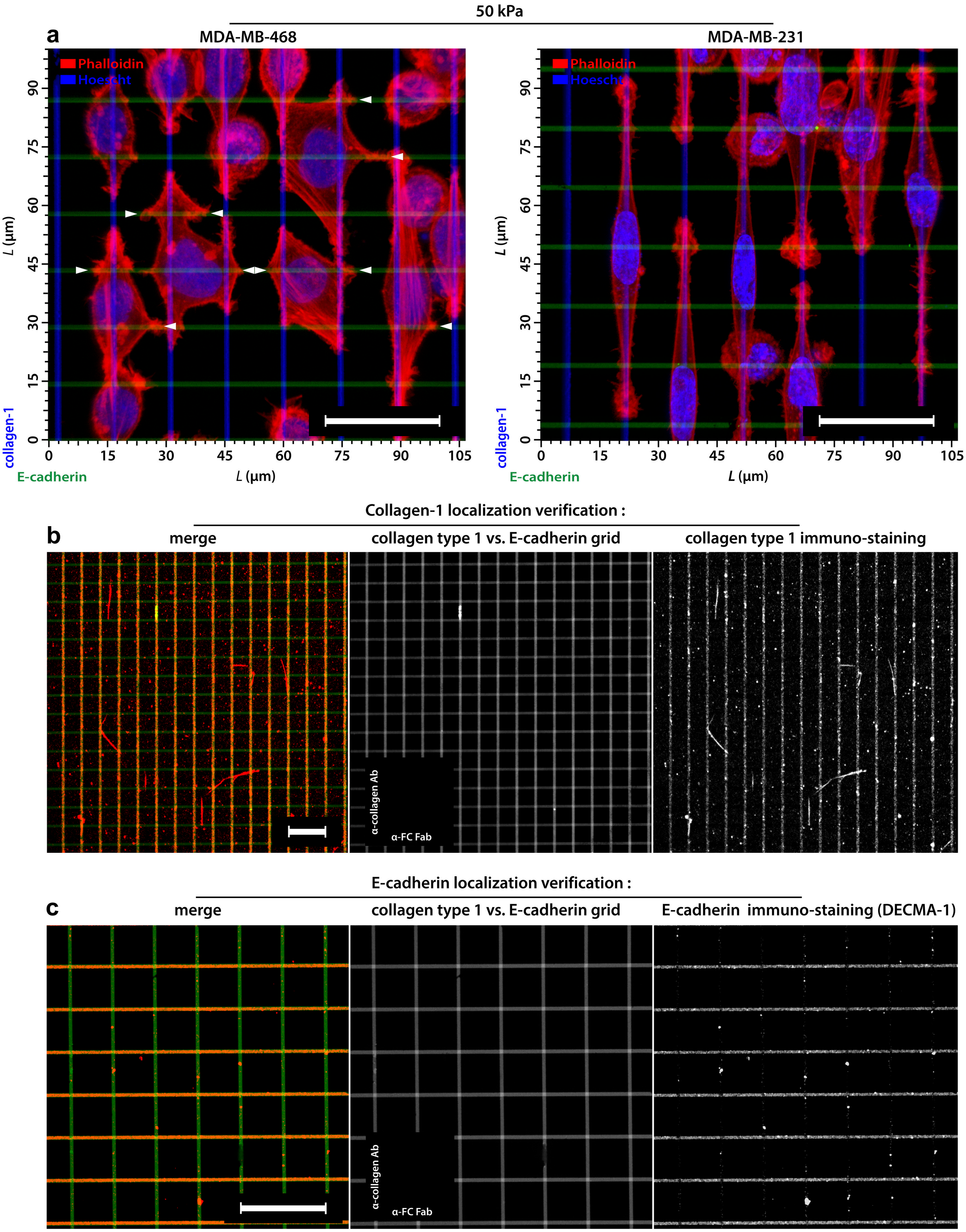
50 kPa
a
MDA-MB-468
MDA-MB-231
Phalloidin
Hoescht
L (µm)
0
15
30
45
60
75
90
105
collagen-1
E-cadherin
Collagen-1 localization verification :
b
merge
collagen type 1 vs. E-cadherin grid
collagen type 1 immuno-staining
α-collagen Ab
α-FC Fab
E-cadherin localization verification :
c
merge
collagen type 1 vs. E-cadherin grid
E-cadherin immuno-staining (DECMA-1)
α-collagen Ab
α-FC Fab

Supplementary Figure 9 | Validation of specificity for type I Collagen and E-cadherin lines

(a) MDA-MB-468 (E-cadherin-positive cells) and MDA-MB-231 (E-cadherin-negative) cells on 50 kPa stiff collagen and E-cadherin orthogonal grids. E-cadherin-positive cells show engagement, adhesion and protrusion along E-cadherin lines, while the E-cadherin-negative MDA-MB-231 cells only engage and spread on collagen-1 lines, and are completely unresponsive to E-cadherin lines confirming that no ECM ligand had absorbed to the lines.

(b) Verification of type I collagen localization with immunofluorescent staining of adsorbed collagen to α-collagen lines (*vertical lines*) within α-collagen-1 and α-FC Fab orthogonal grids. Note that adsorbed collagen is strictly localized at the vertical α-collagen lines.

(c) Verification of E-cadherin-FC chimera protein localization with immunofluorescent staining of E-cadherin extracellular domain (horizontal lines, DECMA-1 Ab) within α-collagen-1 and α-FC Fab orthogonal grids. Note that E-cadherin staining is strictly localized at the horizontal α-FC Fab lines.

Scale bars - 30 μm.

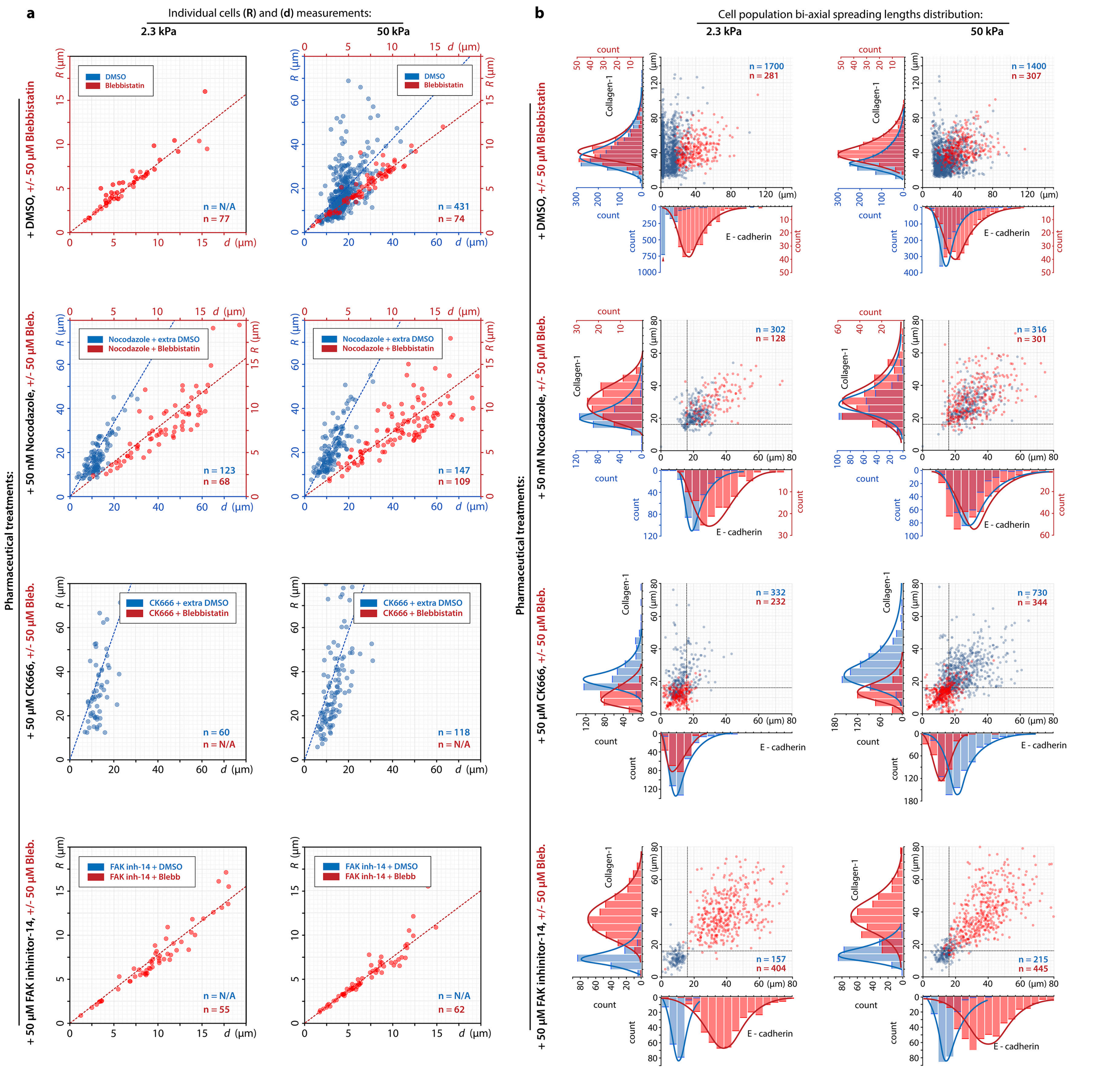

Supplementary Figure 10 | Morphometric and morphomechanical raw data for collagen and E-cadherin cell-adhesion cues recognition

(**a**) Population view of morphomechanical analysis (*R=f(d)*) in contractile cells (*blue*) and in myosin II-inhibited cells (*red*) on soft (2.3 kPa) and stiff (50 kPa) substrates. Corresponding pharmacological treatment conditions are shown in the same order as in panel **a**.

(**b**) Morphometric analysis of cells on competing biaxial collagen vs. E-cadherin cues on soft (2.3 kPa) and stiff (50 kPa) substrates in contractile cells (*blue*) and NM-myosin-II inhibited cells (*red*). Corresponding pharmacological treatment conditions are shown in the following order from top to bottom: +DMSO, +nocodazole, +CK666 and +FAK inhibitor-14. Dashed lines in the plots indicate the region of transition between spread (active protrusion) and non-spread cells (viable cells that do not display robust protrusion). Corresponding n values are shown on the plots. Number of replicates (independent experiments) for all measurements N=5.

Supplementary references

1. Théry, M., Pépin, A., Dressaire, E., Chen, Y. & Bornens, M. Cell distribution of stress fibres in response to the geometry of the adhesive environment. *Cell Motil. Cytoskeleton* **63,** 341–355 (2006).
2. Evangelista, M., Pruyne, D., Amberg, D. C., Boone, C. & Bretscher, A. Formins direct Arp2/3-independent actin filament assembly to polarize cell growth in yeast. *Nat. Cell Biol.* **4,** 32–41 (2002).
3. Fessenden, T. B. *et al.* Dia1-dependent adhesions are required by epithelial tissues to initiate invasion. *J. Cell Biol.* jcb.201703145 (2018).
4. Kozlov, M. M. & Bershadsky, A. D. Processive capping by formin suggests a force-driven mechanism of actin polymerization. *J. Cell Biol.* **167,** 1011–1017 (2004).
5. Shemesh, T. & Kozlov, M. M. Actin polymerization upon processive capping by formin: a model for slowing and acceleration. *Biophys. J.* **92,** 1512–1521 (2007).
6. Hotulainen, P. & Lappalainen, P. Stress fibers are generated by two distinct actin assembly mechanisms in motile cells. *J. Cell Biol.* **173,** 383–394 (2006).
7. Tabdanov, E. *et al.* Micropatterning of TCR and LFA-1 ligands reveals complementary effects on cytoskeleton mechanics in T cells. *Integr. Biol.* **7,** 1272–1284 (2015).
