# Supplementary material for: Bimodal sensing of guidance cues in mechanically distinct microenvironments
Source: Nat Commun. 2018 Nov 20;9:4891. doi: 10.1038/s41467-018-07290-y (PMC6244288; doi:10.1038/s41467-018-07290-y)
Supplement: Supplementary file 4 — Description of Additional Supplementary Files [file 41467_2018_7290_MOESM4_ESM.pdf]

Description of Additional Supplementary Files

File Name: Supplementary Movie 1
Description: MDA-MB-468 cells protrusion dynamics along soft uni-axial type-I collagen lines (+DMSO). Line width is 1 µm, pitch distances between the lines are 15 µm, G'=2.3 kPa.

File Name: Supplementary Movie 2:
Description: MDA-MB-468 cells protrusion dynamics along stiff uni-axial type-I collagen lines (+DMSO). Lines width is 1 µm, pitch distances between the lines are 15 µm, G'=50 kPa.

File Name: Supplementary Movie 3
Description: MDA-MB-468 cells bi-directional protrusion dynamics along stiff bi-axial type-I collagen orthogonal grids (+DMSO). Lines width is 1 µm, pitch distances between the lines are 15 µm, G'=50 kPa.

File Name: Supplementary Movie 4:
Description: MDA-MB-468 cells protrusion dynamics along soft uni-axial collagen type-I lines in 50 µM blebbistatin. Lines width is 1 µm, pitch distances between the lines are 15 µm, G'=2.3 kPa.

File Name: Supplementary Movie 5
Description: Low traction MDA-MB-468 cells dendritic mode of protrusion and migration along soft bi-axial type-I collagen orthogonal grids in 50 µM blebbistatin. Lines width is 1 µm, pitch distances between the lines are 15 µm, G'=2.3 kPa

File Name: Supplementary Movie 6
Description: Low traction MDA-MB-468 cells dendritic mode of protrusion and profound motility along and across stiff bi-axial type-I collagen orthogonal grids in 50 µM blebbistatin. Lines width is 1 µm, pitch distances between the lines are 15 µm, G'=50 kPa.

File Name: Supplementary Movie 7
Description: Low traction MDA-MB-468 cells dendritic mode of protrusion and profound motility along and across soft bi-axial type-I collagen orthogonal grids after disruption of microtubules (50 µM blebbistatin+50 µM nocodazole). Lines width is 1 µm, pitch distances between the lines are 15 µm, G'=50 kPa.

File Name: Supplementary Movie 8
Description: MDA-MB-468 cells protrusion failures (catastrophes) along soft uni-axial type-I collagen lines in 50 µM CK666 Arp2/3 inhibitor. Lines width is 1 µm, pitch distances between the lines are 15 µm, G'=2.3 kPa.

File Name: Supplementary Movie 9

Description: MDA-MB-468 cell steady 1D protrusion along stiff uni-axial type-I collagen lines in 50 µM CK666 Arp2/3 inhibitor. Lines width is 1 µm, pitch distances between the lines are 15 µm, G'=50 kPa.

File Name: Supplementary Movie 10
Description: MDA-MB-468 cell dendritic and dynamic low traction mode of bi-directional protrusion on soft bi-axial collagen type-I orthogonal grid in 25 nM smifH2 formins inhibitor. Lines width is 1 µm, pitch distances between the lines are 15 µm, G'=2.3 kPa.

File Name: Supplementary Movie 11
Description: MDA-MB-468 cell dendritic and dynamic low traction mode of bi-directional protrusion on soft bi-axial collagen type-I orthogonal grid in 25 nM smifH2 formins inhibitor. Lines width is 1 µm, pitch distances between the lines are 15 µm, G'=2.3 kPa

File Name: Supplementary Movie 12
Description: MDA-MB-468 cell steady bi-directional protrusion and subsequent commitment to a uni-axial direction (horizontal cell) on stiff bi-axial type-I collagen orthogonal grids in 50 µM CK666 Arp2/3 inhibitor. Lines width is 1 µm, pitch distances between the lines are 15 µm, G'=50 kPa.
