# Supplementary material for: Bimodal sensing of guidance cues in mechanically distinct microenvironments
Source: Nat Commun. 2018 Nov 20;9:4891. doi: 10.1038/s41467-018-07290-y (PMC6244288; doi:10.1038/s41467-018-07290-y)
Supplement: Supplementary file 3 — Peer Review File [file 41467_2018_7290_MOESM3_ESM.pdf]

Editorial Note: this manuscript has been previously reviewed at another journal that is not operating a transparent peer review scheme. This document only contains reviewer comments and rebuttal letters for versions considered at Nature Communications.

REVIEWERS' COMMENTS:

Reviewer #1 (Remarks to the Author):

I recommend acceptance of the current version of the manuscript in Nature Communications.

RESPONSE TO REVIEWER 1

Reviewer #1 (Remarks to the Author):

I recommend acceptance of the current version of the manuscript in Nature Communications.

Response: We thank the Reviewer for their comment and have edited the manuscript according to the Editor's guidelines to meet the formatting requirements of Nature Communications.
